# Semantic properties and categorization norms for the 260 Snodgrass and Vanderwart objects: A 45-year conceptual update to a classic set

**DOI:** 10.3758/s13428-026-03069-y

**Published:** 2026-07-08

**Authors:** Caitlyn Antal, Roberto G. de Almeida

**Affiliations:** 1 Department of Psychology, McGill University, Montreal, Canada; 2 Department of Psychology, Concordia University, Montreal, Canada; 3 MRC Cognition and Brain Sciences Unit, University of Cambridge, Cambridge, UK

**Keywords:** Property norms, Semantic features, Object concepts, Feature statistics, Object-property congruency task

## Abstract

We present a new set of English property norms for 260 object concepts based on the standardized Snodgrass and Vanderwart (1980) picture set. For each object, 100 participants provided a basic-level label (*dog*), a superordinate category (*animal*), and three features (*bark*, *tail*, *fur*), yielding a dataset of 78,000 features. Our norms differ from other datasets in four important ways: they (1) probe basic-level information, (2) separate taxonomic and feature information, (3) use open-ended responses for natural descriptions, and (4) include a larger number of responses per object (100 vs. ~ 30 participants in other norms). We analyzed feature statistics such as frequency, distinctiveness, and co-occurrence, and contrasted our norms with those of McRae et al. (2005), CSLB (Devereux et al., 2014), and Hovhannisyan et al. (2021). Compared to picture-based norms, our data—derived from black-and-white line drawings—elicited more diverse features and aligned more closely with language-based norms, particularly CSLB. We assessed the generalizability of our norms using an object-property congruency task, where 144 participants judged whether properties (basic level, superordinate, and features) were related to objects. Objects were shown in three picture formats with increasing ecological validity: (1) colored line drawings, (2) realistic photographs, and (3) realistic photographs of objects in scenes. We then contrasted these data with those of Antal & de Almeida (2024) employing the original line-drawing set. Agreement rates for object-property pairs remained high across picture formats and property types. Bayesian inference revealed minimal variability in congruency judgments across picture formats, with responses tightly clustered around zero. Results show that our norms are generalizable to realistic visual stimuli. Norms are available at https://osf.io/c6brw/overview.

**Supplementary Information:**

The online version contains supplementary material available at 10.3758/s13428-026-03069-y.

## Introduction

How is conceptual knowledge represented and processed in the brain? This question is central to cognitive science, as concepts—the basic elements of meaning—underlie core cognitive faculties such as thinking, language, and vision. A common approach to investigating the nature of conceptual representation is the use of property norms, in which participants list features associated with a given concept. These features are then analyzed to identify patterns of co-occurrence and variability across concepts, allowing researchers to infer the structure of representations in semantic memory. However, the methods used to elicit these norms, whether through linguistic labels or pictures, can significantly influence what comes to mind about a concept, thereby impacting the resulting feature sets and corresponding interpretations in theories of conceptual representation.

**Fig. 1:**
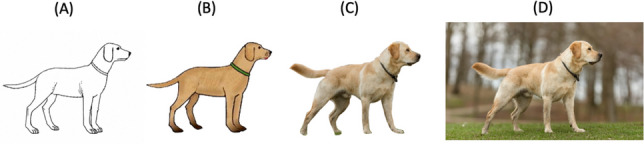
Example of the dog object across the four picture formats: **(A)** black-and-white line drawing from the Snodgrass and Vanderwart (1980) set; **(B)** colored version from the Rossion and Pourtois (2004) set; **(C)** realistic photograph of the object in isolation (background removed); **(D)** realistic photograph of the object embedded in a naturalistic scene. We report norms for (**A**) and also contrast word-picture congruency data from (**A**), obtained from Antal and de Almeida (2024), with data from an experiment involving (**B**), (**C**), and (**D**), reported below

In the present article, we introduce a new set of English property norms elicited from Snodgrass and Vanderwart’s (1980) classic picture set of 260 objects.^1^ For each object, 100 native English speakers provided three types of properties: (1) the basic-level label, (2) the superordinate category label, and (3) three features, yielding a dataset with a total of 78,000 features. We compare our norms with existing datasets, including those from McRae et al. (2005) and the CSLB (Devereux et al., 2014), which used linguistic labels, and Hovhannisyan et al. (2021), which used pictures. This comparison allows us to identify how the method used to probe norming data—whether through linguistic labels or pictures—affects the types of features generated and, consequently, their implications for empirical work as well as for theories on the nature of conceptual representation. Finally, we validate our norms through a series of object-property congruency tasks involving pictures of objects with increasing ecological validity, ranging from black-and-white line drawings to realistic photographs of objects embedded in scenes (see Fig. 1).

### Concepts and property features

Numerous theories have been proposed on the nature and organization of concepts in the brain (see Antal & de Almeida, 2024; Frisby et al., 2023, for recent reviews). For concrete objects, there is widespread agreement that the content of a concept is largely determined by the features of its referent (but see Fodor, 1998; Fodor & Pylyshyn, 2015, de Almeida, 1999, and de Almeida & Antal, 2021, for alternative perspectives). For instance, the concept DOG is often defined by features such as + *bark*, + *fur*, + *tail*, and + *animal* (e.g., Moss et al., 2007; Rogers & McClelland, 2004; Smith & Medin, 1981).^2^ Under this perspective, assigning an object to a particular class (e.g., identifying object X as DOG) relies on computing its properties, particularly its unique and salient features.

Beyond an object’s salient features, feature statistics are thought to be fundamental to conceptual representation as well as conceptual relations. Statistical properties provide crucial information such as a feature’s frequency of occurrence, likelihood of co-occurrence (e.g., + *wings* and + *fly* for BIRD), ontological information type (e.g., motor, sensory, encyclopedic), and distinctiveness or sharedness across concepts (e.g., Cree et al., 2006; Dilkina & Lambon Ralph, 2013; Grondin et al., 2009; Moss et al., 2007; Pexman et al., 2008; Randall et al., 2004; Taylor et al., 2012). For instance, shared features, which occur across many concepts, are thought to be essential for supporting category-level representations (e.g., + *wings* for BIRD), whereas distinctive features, which occur in only one or very few concepts, are important for differentiating between closely related concepts within the same category (e.g., + *udder* for COW).

In order to evaluate the role of feature statistics in the structure and organization of conceptual knowledge, researchers often rely on semantic property norms, which are typically collected through feature-listing tasks. While feature listing tasks are also used in experimental studies to investigate specific phenomena in conceptual processing, such as conceptual combination (e.g., Wu & Barsalou, 2009), norms derived from these tasks have also been central to the development of computational models of semantic memory. In such models, feature statistics are treated as the basic units of conceptual representation (e.g., Rogers & McClelland, 2004; Vigliocco et al., 2004; Cree et al., 2006; Cree & Armstrong, 2012; see also Kumar, 2021, for a recent review). Feature statistics have also been incorporated in AI and cognitive computational approaches, including feature-based embeddings and LLM-assisted datasets (e.g., Derby et al., 2019; Mukherjee et al., 2023; Suresh et al., 2023, 2025). Recent multimodal distributional semantic models, which integrate corpus-based word co-occurrence patterns with property norms, have demonstrated a stronger ability to simulate human behavior across categorization and semantic relation tasks than models relying on either source alone (e.g., Bruni et al., 2014; De Deyne et al., 2021).

In neuroimaging studies, feature statistics have been shown to map onto distinct patterns of brain activity during the perceptual-to-conceptual time course, particularly along the ventral stream (e.g., Bonner & Epstein, 2021; Clarke, 2020; Clarke & Tyler, 2014; Clarke et al., 2013; Cox et al., 2024; Sudre et al., 2012; Tyler et al., 2013). These findings highlight that feature-based information obtained from property norms corresponds to the neural dynamics underlying object recognition and access to object concepts. The development of new norms, based on well-controlled visual stimuli, combined with decoding methods that characterize the representational geometry of these features at the voxel level (e.g., Frisby et al., 2023), will allow researchers to investigate how graded patterns of neural activity evolve along the perceptual-to-conceptual stream.

Feature statistics have also contributed significantly to our understanding of how conceptual knowledge may break down in different neuropsychological populations (e.g., Garrard et al., 2005; Patterson et al., 2007; Lambon Ralph, 2014; Lambon Ralph et al., 2017). For instance, individuals with category-specific semantic deficits, such as the well-documented dissociation between living and nonliving things, often show impairments that reflect the statistical structure of features across domains (e.g., Clarke & Tyler, 2015; Garrard et al., 2001). Living things, which tend to share highly correlated sensory features (e.g., + *eyes* and + *mouth* for ANIMAL), are typically more resilient to damage than nonliving things, which rely more on distinctive functional features (e.g., + *cut* for KNIFE or + *chop* for AXE; see Perri et al., 2012).

Overall, feature statistics are taken to reflect the structure and organization of conceptual knowledge. Property norms provide the necessary data to quantify and analyze these regularities, allowing researchers to investigate the nature of conceptual representation and its breakdown in neuropsychological populations. In the following section, we review existing property norms, highlighting their contributions and limitations, and demonstrate how our norms provide an important and much-needed resource for the scientific community.

### Existing property norms and their limitations

Over the past two decades, researchers have developed a variety of property norms for concrete concepts.^3^ These norms differ along several dimensions, including presentation modality (e.g., linguistic: written words vs. visual: pictures) and methodological approach. Table 1 summarizes widely used property norms developed in English, though many others have been developed in other languages (e.g., Dutch: De Deyne et al., 2008; German: Kremer & Baroni, 2011; Italian: Montefinese et al., 2013; Spanish: Vivas et al., 2017; Chinese: Deng et al., 2021; Portuguese: Valério et al., 2023; Finnish: Kivisaari et al., 2024; see Vivas et al., 2020, for a cross-linguistic comparison), as well as in different populations (e.g., older adults: Vivas et al., 2022; children: Borovsky et al., 2024; blind individuals: Lenci et al., 2013).

**Table 1:** Existing property norms for object concepts in English

Norms	Modality	Stimulus type	Information probed	Items (*N*)	Participants (*N*)	Number of participants per item	Dataset publicly available?
Garrard et al. (2001 )	Linguistic	Concrete objects	Category, Features	64	20	20	No
Randall et al. (2004 )	Linguistic	Concrete objects	Features	93	45	45	No
McRae et al. (2005 )	Linguistic	Concrete objects	Features	541	725	30	Yes
Vinson and Vigliocco (2008 )	Linguistic	Nouns (168 concrete objects), Verbs	Features	456	280	20	Yes
Buchanan et al. (2013 )	Linguistic	Nouns (~ 460 concrete objects), verbs, adjectives, other	Features	1808	2867	66	Yes
Devereux et al. (2014 )	Linguistic	Concrete objects	Features	638	123	30	Yes
Buchanan et al. (2019 )	Linguistic	Nouns (~ 460 concrete objects), verbs, adjectives, other	Features	1914	198	30	Yes
Hovhannisyan et al. (2021 )	Visual	Concrete objects	Features	995	566	30.5	Yes
Present study	Visual	Concrete objects	Object name, Category, Features	260	100	100	Yes

One of the first systematic collections of property norms in English was conducted by Garrard et al. (2001), who collected norms for 64 concrete nouns, each representing an object concept from the Snodgrass and Vanderwart (1980) picture set. In their study, 20 participants were given booklets, with each page focused on a concrete noun (e.g., *elephant*). For each noun, participants completed sentence stems (e.g., “an elephant is __”, “has __”, “can __”), probing category information and six properties of the item. In a similar study, Randall et al. (2004) collected property norms for 93 concrete nouns, spanning both living and nonliving categories. Forty-five participants completed a feature-listing task, in which they were asked to generate as many properties as possible for each item. The datasets from Garrard et al. (2001) and Randall et al. (2004) were among the first to investigate the distinction between sensory and functional features, as well as the role of feature distinctiveness and correlational strength across living and nonliving categories. However, neither dataset is publicly available, limiting their use for large-scale modeling and replication studies.

Thus far, the most comprehensive and publicly available property norms in English are those collected by McRae et al., (2005; see also Cree & McRae, 2003) and by the Center for Speech, Language, and the Brain (CSLB; Devereux et al., 2014). In their study, McRae et al. conducted a feature-listing task for 541 concrete nouns, which included both living and nonliving entities. Data were collected in three phases and across different institutions, involving a total of “approximately 725 participants” (p. 547). Participants received booklets containing 20 to 24 nouns, with ten blank lines per noun, and were instructed to generate as many features as they could for each item. Participants were encouraged to list features describing various types of properties, including physical attributes (e.g., *has fur*), functional properties (e.g., *used for cutting*), behavioral characteristics (e.g., *barks*), as well as other properties such as taxonomic information (e.g., *is an animal*) and encyclopedic knowledge (e.g., where *x* is from). Building on McRae et al.’s (2005) work, the CSLB (Devereux et al., 2014) collected norms for an additional 97 concrete nouns, resulting in a dataset of 638 concepts. In their study, 123 participants completed an online feature-listing task, where they were presented with 30 object concepts and asked to generate at least five properties for every concept. Each trial displayed the object label (e.g., *zebra*) along with blank spaces for listing features. Participants were prompted to select a relation word from a dropdown menu (e.g., *is*, *has*, *does*, *made of*), helping guide them in describing physical attributes, functional properties, and behavioral characteristics of the object concept. Unlike McRae et al.’s dataset, which only includes features produced by at least five participants, the CSLB norms include all features produced by at least two participants, providing researchers with greater flexibility in setting their own cutoff points for feature inclusion. Together, these datasets offer a range of feature statistics for concrete objects, such as production frequency, feature distinctiveness, cue validity, and intercorrelational strength. These measures have been extensively used in computational models and empirical studies on semantic memory (e.g., Clarke, 2015, 2020; Clarke & Tyler, 2014; Clarke et al., 2013, 2018; Tyler et al., 2013).

Beyond concrete nouns, several studies have extended property norms to also include other lexical categories. For instance, Vinson and Vigliocco (2008) were the first to collect property norms for events, ranging from simple actions (e.g., *kicking*) to abstract events (e.g., *exchanging*); see also Vigliocco et al. (2004). In their study, 280 participants generated features for 456 words, including 169 nouns referring to concrete objects (e.g., *apple*), 71 nouns referring to events (e.g., *blink*; “the blink of an eye”), and 216 verbs referring to events (e.g., *throw*). Each participant provided features for a subset of 30 to 40 words and was instructed to avoid free associations and “dictionary-style definitions” (p. 187). In two subsequent studies, Buchanan et al., (2013, 2019) expanded property-norm collections to include not only nouns and verbs, but also adjectives, adverbs, pronouns, prepositions, and interjections. In both studies, participants completed a feature-listing task based on the instructions used by McRae et al. (2005). Specifically, participants were asked to list properties for each word, including its physical, functional, and categorical characteristics. The first study (Buchanan et al., 2013) collected norms for 1808 words from 2867 participants, with each participant contributing properties for an average of 66 items. The second study (Buchanan et al., 2019) expanded the dataset to 1914 words, with 198 new participants providing norms for a subset of 30 items.

All the norms discussed up to this point have been derived from linguistic stimuli, where participants generate features based on word labels. While this approach has allowed researchers to investigate the role of feature statistics on the representation and organization of conceptual knowledge, the properties accessed through linguistic labels may differ from those accessed through the visual modality, such as pictures of objects. This distinction in modality is important because concepts are not solely accessed through language. Visual information also plays a crucial role in how we recognize, categorize, and interact with objects in the real world. As a result, relying exclusively on norms derived from linguistic stimuli may overlook important visual features central to conceptual representation, underscoring the need for complementary norms obtained through the visual modality.

Thus far, only one set of norming data has been collected from the visual presentation of objects (Hovhannisyan et al., 2021). In this study, 566 participants completed a feature-listing task involving 995 objects. Each participant viewed 40 pictures of objects and was instructed to list five features per object. For each feature, participants selected a relation word from a dropdown menu (e.g., *is*, *has*, *does*, *made of*) to encourage a diverse range of object properties. On average, each object was normed by 30 participants.

Although Hovhannisyan et al. (2021) were the first to collect properties and compute feature statistics for objects using pictures, their norms differ from other property norming studies in several important ways. Participants each generated properties for 40 objects, yielding a final dataset of 5,520 unique normalized feature labels, with only features produced at least three times retained in the final norms (p. 718). By comparison, the McRae et al. (2005) norms contain approximately 2,526 unique feature labels and the CSLB (Devereux et al., 2014) norms contain approximately 2,725 unique feature labels. The larger number of unique feature labels in Hovhannisyan et al. may reflect greater variability in the types of properties participants generated across objects and images. 

Another point to be highlighted about Hovhannisyan et al.'s (2021) norms relates to the visual specificity of the stimuli they employed. While many of the pictures they used were appropriate for eliciting object features, a large subset depicted objects embedded in rich visual contexts or scene-like configurations. This degree of specificity does not imply that the resulting features are incorrect, nor that contextual information is conceptually irrelevant. Rather, such stimuli could introduce ambiguity regarding whether the elicited features reflect conceptual properties of the target object or properties of extraneous elements depicted in the picture, in addition to raising questions about the level of representation indexed by the norms (e.g., basic level [BASKET] vs. subordinate level [TOY BASKET]).

For instance, the picture for TOY BASKET depicts a basket overflowing with toys. In this case, TOY functions as a modifier specifying the type of basket (i.e., a basket for storing toys), instead of shifting the reference to TOY as the target concept. However, participants’ responses predominantly describe the contents of the picture (e.g., + *has toys*, + *does hold toys*, + *is full of toys*), and the category label assigned to the item is “TOYS”. Thus, the generated features seem to reflect what is perceptually present in the picture rather than the properties of the object concept denoted by the label (TOY BASKET). Similar visually specific, context-heavy depictions appear for several other items (e.g., DISHWASHER, EASTER EGG, REFRIGERATOR, SHRIMP DINNER, FIREWOOD, GAS STATION), where features may reflect elements of the context instead of object properties that generalize across visual instantiations.

Taken together, the Hovhannisyan et al. norms may not clearly distinguish object-level conceptual properties from contextually cued information introduced by visually cluttered or highly specific stimuli, making it difficult to isolate the conceptual features of the target object. While such norms may be well suited for investigating representations tied to specific exemplars or usage contexts, they are less optimal for characterizing conceptual features of objects independent of incidental visual context. This limitation, therefore, concerns the scope and interpretability of the norms *qua* measures of features taken to constitute the mental representations of an object concept, not the validity of the features themselves.

While existing property norms have significantly advanced our understanding of the nature and organization of concepts in the brain, they, too, are not without limitations. For one, many studies rely on drop-down menus to guide responses, which can constrain participants and lead to unnatural or incomplete feature descriptions (e.g., *does make choice* for MENU [Devereux et al., 2014]; *do protect feet* for BOOTIES [Hovhannisyan et al., 2021]). Additionally, “feature extraction” methods often produce inconsistent units of analysis due to “feature-splitting rules”, which can lead to redundancy (Devereux et al., 2014, p. 1122). For instance, adjectival compounds such as “*has a long neck*” may be split into “*long neck*” and “*neck*,” rather than reduced to the head noun “*neck*”. This is based on the assumption that “*long-necked*”, a property attributed to giraffes, specifically predicates something about their necks. Furthermore, no study to date has systematically and separately probed basic-level labels, superordinate category labels, and object features. In current norming paradigms, participants are typically given the basic-level label (e.g., the name of the object) and instructed to generate features. However, participants often include taxonomic information (e.g., superordinate category information such as “*animal*” or “*vehicle*”) in their feature listings. This information is subsequently removed, as taxonomic features are not generally regarded as true conceptual features (see Smith & Medin, 1981; McRae et al., 2005; Taylor et al., 2012). Moreover, basic-level labels are rarely collected independently. Most norming studies, regardless of modality, present participants with the basic-level label directly. Therefore, it is crucial for a norming study to separately collect basic-level labels, superordinate category labels, and features associated with object concepts in order to be able to assess the impact of hierarchical categorical information.

Beyond these issues, although some datasets include large sample sizes and a broad range of items, they often suffer from a low number of responses per item, limiting the robustness of the data (see Table 1). For instance, McRae et al. (2005) collected data from 725 participants across 541 items, but each item was normed by only 30 participants on average. Similarly, the CSLB norms (Devereux et al., 2014) gathered responses from 123 participants for 638 items, again averaging 30 responses per item. Even in larger datasets, such as Buchanan et al. (2013), which included 1808 items and 2867 participants, the mean number of participants per item was 66. This is a relatively low number given the scale of the dataset. A limited number of responses per item can reduce the reliability and generalizability of the resulting norms, particularly for less common and more complex concepts. This issue is compounded by the fact that each study applies its own feature production frequency cutoff, which varies considerably. For instance, some studies required that a feature be produced by at least two participants (e.g., Devereux et al., 2014), others used a threshold of three (e.g., Hovhannisyan et al., 2021), while McRae et al. (2005) required a minimum of five responses per feature. In other cases, features produced by fewer than 2% of participants were excluded, which corresponded to roughly two to five responses per concept (e.g., Buchanan et al., 2013, 2019). These varying cutoff thresholds further reduce the number of retained features, thereby significantly constraining the final dataset.

Our study addresses these key limitations in existing norms and provides a complementary data source to the earlier datasets. First, our norms are derived from visual stimuli, ensuring that features reflect properties accessed through real-world object recognition rather than purely verbal associations. This is particularly important given the central role of visual information in object categorization and conceptual access. Second, we systematically and separately collect basic-level labels, superordinate category labels, and object features, and thus avoid conflating taxonomic information with conceptual features. This methodological approach also provides a framework for investigating category membership, feature-based representations, and the distribution of features across categories. Third, our norms overcome the issue of unnatural feature descriptions associated with drop-down menus by allowing participants to generate features freely, without predefined relation words. Finally, our norms address the limited number of responses per item found in existing datasets. With 100 participants per object, our dataset contains more responses per concept than any property norming study to date. This far exceeds the typical average of 20 to 66 participants per item reported in the other norms (e.g., Garrard et al., 2001; McRae et al., 2005; Devereux et al., 2014; Buchanan et al., 2013, 2019; see Table 1). The large sample size per item in our norms also allows for potentially more robust statistical analyses and reduces the likelihood of excluding meaningful, albeit less frequently produced, features due to arbitrary production frequency cutoff thresholds.

### The present study: Conceptual features from objects

The goal of the present study was to introduce a new set of features and categorization norms based on Snodgrass and Vanderwart’s (1980) standardized picture set of 260 object concepts. For each object, 100 participants generated (a) the basic-level label, (b) the superordinate category label, and (c) three features about the object, using one word per response. Since every participant responded to all 260 objects, each object was normed by 100 participants, resulting in a dataset of 78,000 features, in addition to the basic-level and category labels. These data, along with various category and feature statistics (see section Description of normed variables), are available in the OSF repository for the present article. We also provide the raw responses, allowing researchers to apply their own response-cleaning criteria and production-frequency cutoffs. Together, the category labels and features we collected make the present norms uniquely suited for investigating the structure of conceptual knowledge from visual input, enabling researchers to systematically manipulate and analyze category information, conceptual features, and their distribution across a set of standardized, well-controlled visual stimuli.

We selected the Snodgrass and Vanderwart picture set because it is one of the most widely used materials in psycholinguistics and cognitive neuroscience experiments. It has served as the basis for studies on language (e.g., Beyersmann et al., 2024), memory (e.g., O’Donnell et al., 2018; Gerlach et al., 2023), and conceptual processing (e.g., Antal & de Almeida, 2024; Hoffman & Lambon Ralph, 2018) across diverse populations, including healthy adults, children, and individuals with brain damage (e.g., Patterson et al., 2007; de Almeida et al., 2021; Kandel & Snedeker, 2024; Henderson et al., 2025). This picture set has also been adapted into multiple languages (e.g., French: Alario & Ferrand, 1999; Italian: Dell’acqua et al., 2000; Icelandic: Pind et al., 2000; Spanish: Fernández et al., 2004; Chinese: Yoon et al., 2004; Russian: Tsaparina et al., 2011). Despite being developed over 45 years ago, the Snodgrass and Vanderwart norms have stood the test of time. They have been cited over 7500 times, including approximately 4000 citations in the last 10 years, as of the writing of the present article. The images have since been updated to include grayscale texture, surface details, and color information (Rossion & Pourtois, 2004). While the original norms provided measures such as name agreement, image agreement, familiarity, and complexity, they did not include information about object properties. Our norms substantially extend the utility of the Snodgrass and Vanderwart set by providing categorical and feature-based data for all 260 object concepts, allowing researchers to develop materials that take into account an object’s category membership and its distribution of features. Our norms also allow for the reanalysis of existing studies as a function of categorization and feature statistics.

There are several additional reasons for basing our norms on the Snodgrass and Vanderwart picture set. One such reason is that line drawings have been shown to be as effective as colored photographs in triggering object properties (e.g., Walther et al., 2011). In particular, line drawings tend to elicit more accurate object categorizations when stimuli are presented briefly (e.g., 13 ms; Fu et al., 2016). A second and more important reason is that shape- and edge-based information, rather than color information, have been shown to facilitate the decoding of objects during the early moments of object processing (e.g., Yao et al., 2023; see also Biederman, 1987; Biederman & Ju, 1988).^4^ A third reason is that using line drawings allows us to elicit color information that would otherwise be unavailable in the colored version of the picture set. A fourth reason is that line drawings are void of visual clutter, ensuring that the properties listed by participants reflect the conceptual features of the target object, rather than those of extraneous items in the picture. Finally, the Snodgrass and Vanderwart line drawings are more easily modified than colored images, making them well suited for a variety of research paradigms (e.g., De Winter & Wagemans, 2004, 2008; Magnié et al., 2003). For instance, in Antal and de Almeida (2024), we modified the color of the black lines in the Snodgrass and Vanderwart drawings to match the hue of red-blue anaglyph glasses. This allowed us to control for overlapping retinal projections and independently project stimuli to the left- and right-hemisphere visual areas, while participants completed a semantic congruency task using dichoptic presentations of pictures and words. Other studies have created chimeric objects by combining parts of different objects in the Snodgrass and Vanderwart picture set (e.g., Gerlach et al., 2023; Kahlaoui et al., 2007). By collecting norms on their line drawings, our dataset remains adaptable to different experimental manipulations, making it a more versatile resource for studies that require customized stimuli.

In addition to introducing a new set of property norms, we conducted a behavioral experiment to determine whether the features and category labels elicited from black-and-white line drawings generalize to visual stimuli with greater ecological validity. The experiment used an object (picture)-property (word) congruency task in which participants judged whether an object property, such as a basic-level label (e.g., *dog*), a superordinate label (e.g., *animal*), or a high- or low-salient feature (e.g., *bark*, *fur*), was related to an object depicted in a picture (e.g., dog). The same objects were contrasted relying on four increasingly naturalistic visual formats: (1) the original black-and-white line drawings from Snodgrass and Vanderwart (data from Antal & de Almeida, 2024, were included for comparisons), (2) colored versions of the line drawings, (3) realistic photographs of objects in isolation, and (4) photographs of objects embedded in real-world scenes. The last three sets were the stimuli for the experiment reported below.

The experiment served two purposes. First, it evaluated the generalizability of the norms by determining whether the same conceptual information is accessed when objects are shown with color, texture, and in context. Second, it demonstrated how these norms can be used in experimental paradigms to study conceptual processing across different visual stimuli. Unlike tasks that emphasize lexical access (e.g., picture naming, lexical decision), the picture-word congruency judgment paradigm probes access to an object concept and its associated properties. As reported below, in Sect. 5, congruency judgments were highly consistent across picture formats, providing support for the generalizability of the norms across picture formats varying in ecological validity.

In the following sections, we describe our norms and compare them to established datasets, such as those from McRae et al. (2005) and the CSLB (Devereux et al., 2014), which were collected from linguistic labels, and Hovhannisyan et al. (2021), which were collected from pictures. This comparison allowed us to investigate how the method of data collection, whether through words or pictures, affects the types of properties generated about a concept. We conclude by assessing the generalizability of our norms, using the object-property congruency task with stimuli of increasing ecological validity, to test whether the conceptual information elicited from line drawings holds across more naturalistic visual inputs. Overall, the following analyses will demonstrate that the norms collected here provide a unique and valuable tool for designing experiments on object recognition and semantic memory, as well as new training materials for computational models.

## The norms

### Method

#### Participants

We recruited 100 participants from Amazon’s Mechanical Turk (AMT; *M*_age_ = 39, *SD*_age_ = 12). All participants were native speakers of English (i.e., learned English before the age of 5) and used it as a dominant language. Participants were required to have an approval rate of at least 80% and be located in one of the following countries: Australia, Bahamas, Barbados, Canada, Ireland, Jamaica, New Zealand, the United Kingdom, the United States, and the Virgin Islands (USA and British). Of the 100 participants, 98 were from the USA, and two were from Canada. Each participant received monetary compensation for completing the survey (USD 8.46 per hour of completed work). All participants gave informed consent and were treated in accordance with the ethical standards adhered to by Concordia University’s Human Research Ethics Committee.

#### Stimuli and procedure

**Fig. 2:**
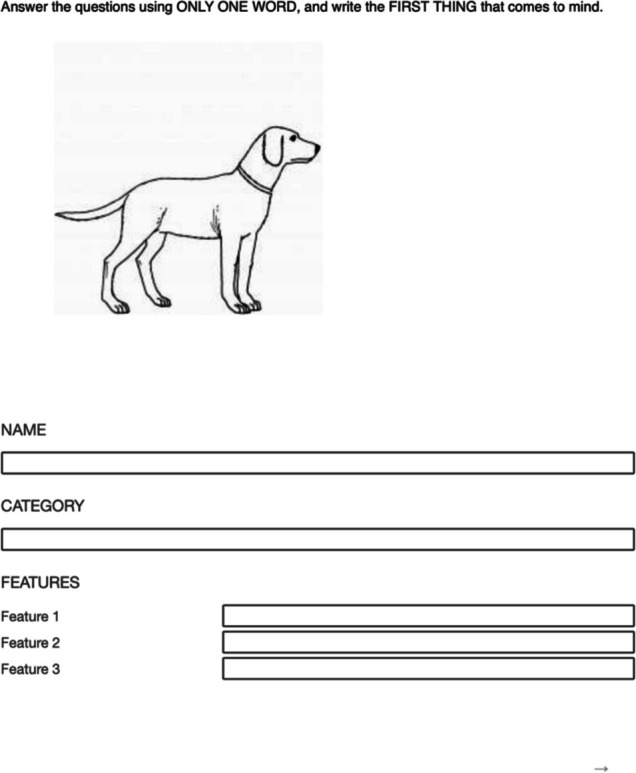
Example trial depicting the “dog” picture

The stimulus set included all 260 standardized pictures developed by Snodgrass and Vanderwart (1980), with 99 depicting living objects and 161 nonliving objects. The norming task was administered via Qualtrics (2015), which was embedded within the AMT platform. The survey first presented participants with the consent form and a short language background questionnaire. The language background questionnaire assessed participants’ self-reported language history and proficiency. Once participants completed the consent form and the language background questionnaire, they were presented with the general instructions of the norming task. Participants were instructed that they would be presented with pictures of objects, one at a time, and that for each picture, they would need to complete three tasks (see Fig. 2 for an example trial). They were first instructed to provide the name of the object in the picture, as briefly and unambiguously as possible, targeting basic-level labels. Then, they were instructed to categorize the object in general terms, targeting superordinate-level labels. And lastly, participants were instructed to list three features that came to mind about that object. Examples of physical, functional, sensory, and behavioral features of the objects were provided to illustrate the range of possible responses, but participants were not restricted to these types of features. For all three questions, participants were asked to respond using only one word or a hyphenated word combination and to use the first word that came to mind. Participants were presented with all 260 pictures, in random order, and at a reduced size of 500 × 500 pixels. The norming task began with three example trials and took approximately 2 h to complete.

### Data cleaning criteria

We first screened participants’ responses for compliance with instructions. Participants submitting incomplete surveys or surveys with non-compliant responses (e.g., “*NA”, “none”, “…”, or responses that included more than one word*) were rejected without payment. This led to the removal of 16 participants, yielding a final sample of 100.

After removing non-compliant participants, we sorted all responses across the 260 objects into three files, one for each of the tasks: (1) object naming responses, (2) category naming responses, and (3) object feature responses. In order to systematize the data cleaning procedure, we applied the same criteria across each task. We also corrected obvious spelling errors (e.g., *insturment* became *instrument*, *acesory* became *accessory*) and removed non-sensical or uninterpretable responses (4 responses in total: *of* as a feature for the item *gun*, *takic* as a feature for the item *deer*, *ggel* as a feature for the item *horse*, and *fulful* as a feature for the item *sandwhich*).

We then decomposed responses into their morphological forms using the WebCelex wordform database, aiming to narrow down responses to the most common morpheme (e.g., *animals* became *animal*, *hooves* became *hoof*, *addiction* and *addictive* became *addict*). Certain complex responses, such as existing compounds, were left intact, for they more appropriately represented the referent object (e.g., *airplane* was not reduced to *plane* and *body part* was not reduced to *part*). Novel compounds, however, were reduced to their compound heads (e.g., *pet accessory* was reduced to *accessory*; *sport vehicle* was reduced to *vehicle*). In some cases, the modifier was used instead of the head, for it better represented the item (e.g., *light source* became *light*, which was the dominant category response for the item *candle*).

Adjectival compounds (e.g., *long-necked*, *long-eared*) were reduced to their heads. We reasoned that *long-necked* for instance, a property attributed to *giraffe,* was an attempt to predicate at the *neck* of giraffes, thus making that particular referent salient. We took a similar approach with compounds such as *old-fashion(ed)*, *butterknife*, and *beachball*; in these cases, we attempted to preserve content by reducing the compounds to the modifier, yielding *old*, *knife,* and *ball*, respectively.

There were other cases in which morphological reduction was not possible, as this yielded the wrong form of predication. For instance, negations were preserved to maintain the correct predication (e.g., *nonliving ≠ living, pointless ≠ point*). We only converted a negation to its lexical equivalent in one case (*not bad* became *good*). We did this on the assumption that removing the negation, in this case, would yield the opposite of the participant's intended response.

We made several other changes to align with the most commonly provided answers. This implied modifying cases of derived words for other, more common, derivations. For instance, for the item *pen*, the category name most provided was *writing*; therefore, one instance of *writer* was also changed to *writing* to reflect the common category name provided. We also reduced responses that were infrequent (i.e., non-dominant) to the most commonly listed response represented by the morphological root (e.g., *signal*, *signage*, *signs* all became *sign*, which was the dominant category response for the item *arrow*). This criterion did not apply to cases in which there were several frequent responses, even when these responses had a common morpheme, or commonly attributed responses (e.g., *airplane* and *plane* were both left intact for the item *airplane*; *bike*, *bicycle*, and *cycle* were also left intact for the item *bicycle*).

Lastly, inflected responses were reduced to the most common stem (e.g., *drugs* reduced to *drug*), but derivations were not (e.g., *smoke* and *smoking* were kept as such; notice that *-ing* can also serve as inflection, but for the most part, we had no way to distinguish their uses). There were also exceptions to this criterion, when, for instance, the plural form better named the object (e.g., *lips* was not reduced to *lip*). There were cases in which the plural form was the most commonly used, and that was also kept intact (e.g., *toiletries* was not changed to *toiletry,* which works only as a modifier; *eyeglasses* was not changed to *eyeglass*). Participants’ raw responses can be found in the Excel file we have uploaded on OSF, particularly, in the tab entitled “Features-Raw”.

## Description of normed variables

In the present section, we describe the measures obtained from the norms and the statistical analyses conducted on these norms. The norms, data files, and scripts for all analyses are available on OSF (https://osf.io/c6brw/overview). This study was not preregistered.

We organized the norms across multiple tabs in an Excel file, each reporting different statistical measures on object concepts. The first tab, labeled “Measures-Explanations,” serves as a reference guide, providing definitions and methodological details for all measures included in the norms. In the following sections, we describe the information contained in each subsequent tab of the Excel file. Detailed information about the organization of the norms and data files can be found in the readme file on OSF.

### Category and object naming

Information on category and basic-level object naming for each picture is found in the second and third tabs of the Excel file, labeled “Superordinate-Level-Labels” and “Basic-Level-Labels,” respectively. The column names in these tabs report the following measures:

- **Domain**: Indicates whether the object belongs to the domain of living or nonliving things.
- **Item picture**: The name of the object concept depicted in the picture, as originally reported by Snodgrass and Vanderwart (1980).
- **Superordinate- and basic-level label**: Lists all names, including alternative names, generated for each picture. The “Superordinate-Level-Labels” tab includes category (i.e., superordinate) labels, while the “Basic-Level-Labels” tab contains all object (i.e., basic-level) labels.
- **Production frequency:** Reports the number of participants who produced a name for a picture, listed in descending order of frequency. The values represent production frequency scores expressed as percentages, based on responses from 100 participants.
- ***H*-statistic:** Reports the information-theoretical *H*-statistic (or “entropy”) for each picture, quantifying the uncertainty among participants regarding the name attributed to a picture. This measure has been shown to be a stronger predictor of naming latencies than simple name agreement (e.g., Snodgrass & Vanderwart, 1980; Székely et al., 2003). We computed this measure following Shannon (1948) and Snodgrass and Vanderwart (1980), and report it for both basic- and superordinate-level responses:
  $$H= \sum_{i=1}^{k}{p}_{i}{\mathrm{l}\mathrm{o}\mathrm{g}}_{2}\left(1/{p}_{i}\right)$$

In this equation, *k* represents the number of different responses given for each picture, and *Pi* represents the proportion of participants who produced each response. Low *H*-statistic values indicate minimal uncertainty among participants, signaling a high consensus in naming (e.g., an *H*-statistic of 0 suggests unanimous agreement). Conversely, high *H*-statistic values indicate increased uncertainty, reflecting considerable diversity in naming.

(f) **Word length:** Reports the number of letters for each final spelling-corrected category and object name provided for a given picture (see Data cleaning criteria section).
(g) **Social-usage word frequencies:** For each superordinate and basic-level object label, we report various measures of social-usage word frequencies, including: (a) word frequency (WF), (b) contextual diversity (CD), discourse contextual diversity (DCD), and user contextual diversity (DCD). We also included derivatives of the UCD and DCD measures at the population (i.e., PR) and word level (i.e., WR), which consider the semantic diversity of contexts in which words occur (Johns, 2021; see also Hoffman et al., 2013; Chapman & Martin, 2022), with words that appear in more unique semantic contexts receiving a higher strength in memory. These variables were derived from Johns (2021), who used a database of Reddit comments containing over 55 billion words. Word frequency is the number of times a word occurs in the text. DCD is the number of different discourses (subReddits) in which a word occurred. UCD is the number of individual users who produced the word. We included these social-usage frequency measures in our norms because they have been shown to outperform traditional count-based frequency measures in a variety of behavioral studies on conceptual access (e.g., picture-word congruency task: Antal & de Almeida, 2024; Antal et al., 2025) and lexical access (e.g., lexical decision tasks: Antal et al., 2026; spoken word recognition: Antal & de Almeida, 2023).

### Object features

Object feature information is reported in tabs four to six of the Excel file: “Final-Features-Cleaned”, “Norms-PFA”, and “Norms-PF5”. The content and organization of these tabs are described below.

The “Norms-PFA” tab includes production frequency data for all features, while the “Norms-PF5” tab includes only features with a production frequency of at least 5. We use this cutoff value solely for comparability with prior norms, as it was also employed by McRae et al. (2005) and the CSLB norms (Devereux et al., 2014). Crucially, we also provide information about all features in the “Norms-PFA” tab, allowing researchers to apply their own production frequency cutoff points if desired. Low-frequency features (e.g., those produced by a single participant) are not treated as characteristic of object concepts and were excluded from all descriptive and comparative analyses reported here; they are retained only to preserve the completeness of the raw data.

#### Final features cleaned

The “Final-Features-Cleaned” tab contains all features generated by participants, with spelling corrections applied. Each column header corresponds to the name of the object depicted in the picture, and entries within these columns represent the features generated by participants for that object. A total of 300 features are listed for each of the 260 objects, resulting in a dataset of 78,000 features across all pictures.

#### Norms-PFA

The “Norms-PFA” tab provides information about the feature distribution for each object. For convenience, we included measures we reported in Sect. 3.1, such as “domain” and “item picture”. We also report the social-usage frequency associated with each feature label. The remaining columns report the following measures:

- **Basic-level concept:** Indicates the basic-level label most frequently listed for a given picture. This label corresponds to the basic-level name with the highest production frequency in the “Basic-Level-Labels” tab (see section Category and object naming).
- **Basic-level concept production frequency:** Reports the production frequency of the basic-level label most frequently listed for a given picture. Since each picture has 100 responses, this value can also be interpreted as a percentage.
- **Superordinate category:** Indicates the superordinate-level label most frequently listed for a given picture. This label corresponds to the superordinate level name with the highest production frequency in the “Superordinate-Level-Labels” tab (see section Category and object naming).
- **Superordinate category production frequency:** Reports the production frequency of the superordinate-level label most frequently listed for a given picture. Since each picture has 100 responses, this value can also be interpreted as a percentage.
- **Feature name:** Lists all features generated for a given picture.
- **Feature sensorimotor dominant:** Specifies the dominant sensorimotor modality of the feature name, based on the Lancaster Sensorimotor Norms (Lynott et al., 2020).
- **Feature production frequency:** Reports the number of participants who produced a given feature for a picture, listed in descending order of frequency.
- **Feature occurrence:** Indicates the number of objects from the norms in which a given feature occurs.
- **Feature total production frequency:** Indicates the number of times a given feature is produced across all 260 pictures.
- **Feature distinctiveness:** Indicates how unique or shared a feature is for a particular object. It is defined as the inverse of the total number of pictures in which a given feature appears across the dataset (i.e., 1/[feature occurrence]). Thus, distinctiveness ranges along a continuum, from high distinctiveness (i.e., features unique to an object) to low distinctiveness (i.e., features shared across objects). We included this measure in our norms because feature distinctiveness is a key factor in concept theories (e.g., Conceptual Structure Account; Moss et al., 2007). Additionally, research has shown that object concepts with more distinctive features tend to have shorter recognition and naming latencies (e.g., Devereux et al., 2016; Taylor et al., 2012).
- **Feature cue validity (CV):** Calculated by dividing the production frequency of a given feature for a particular object by the total production frequency of that feature across all 260 pictures (i.e., feature_prod_freq/feature_total_prod_freq; e.g., Rosch & Mervis, 1975; Reed, 1972).

#### Norms-PF5

In addition to adapting the information reported in the “Features-PFA” tab to include only features with a minimum production frequency of five, we report on a variety of feature-based statistical measures that focus on the relational structure of features within a concept. These measures include feature co-occurrence strength, sharedness, and distinctiveness. We included these measures because they are central to many feature-based theories of concepts and have been shown to play a key role in the representation and processing of object concepts (e.g., Hovhannisyan et al., 2021; Moss et al., 2007; Taylor et al., 2012). Furthermore, these measures are crucial for explaining patterns of category-specific semantic deficits observed in neuropsychological patients with various aetiologies, such as the well-documented dissociation between living and nonliving things (e.g., Clarke & Tyler, 2015; Dilkina & Lambon Ralph, 2013). These feature statistics were also included to allow for comparisons with existing property norms, which require that a minimum production frequency cutoff of five be applied (e.g., McRae et al., 2005; CSLB: Devereux et al., 2014; Hovhannisyan et al., 2021).

**Fig. 3:**
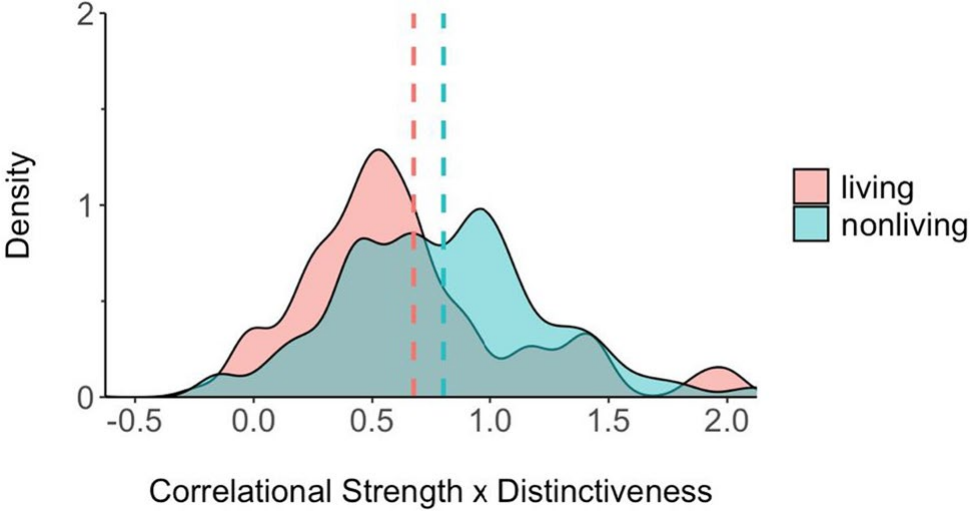
Density plots showing the distribution of the correlational strength *x* distinctiveness scores as a function of the objects’ living/nonliving distinction. The *dashed lines* represent the mean

- **Concept number of features:** Indicates the number of distinct features generated for a concept, regardless of whether they are shared with other concepts. For example, if *bark* is listed three times, it counts as one unique feature.
- **Number of distinctive features per concept:** Indicates the number of features listed for a concept that occur in only one or two object concepts in the norms.
- **Mean feature distinctiveness per concept:** Reports the average distinctiveness of all features associated with an object concept.
- **Number of shared features per concept:** Reports the number of features in a concept that occur in three or more object concepts in the norms.
- **Mean correlational strength by feature:** This feature-level measure reports the average of all significant Pearson correlation coefficients between a target feature and all other features in the norms, indexing how often features co-occur across concepts. This measure reflects feature sharedness and has been shown to support faster conceptual processing (e.g., McRae et al., 1997; Taylor et al., 2012) and to predict neural activity in the ventral stream, particularly in regions implicated in category structure (e.g., Clarke, 2015; Tyler et al., 2013). Following McRae et al. (2005) and Taylor et al. (2012), correlations were computed from a concept-by-feature matrix (concepts in rows, features in columns), with cell values reflecting production frequency. Features occurring in only one or two concepts were excluded, and cells with “#N/A” indicate no significant correlations. The matrix is available in the “Feature-Correlation-Matrix” tab.
- **Number of significant correlations by feature:** Reports the number of statistically significant correlated feature pairs that include a target feature in the norms.
- **Correlational strength by distinctiveness:** This concept-level measure, based on Taylor et al. (2012), quantifies the structural relationship between features within a concept. Specifically, it reflects how feature co-occurrence (correlational strength) relates to feature uniqueness (distinctiveness) for a given object, calculated as the unstandardized slope of a regression line with correlational strength on the *x*-axis and distinctiveness on the *y*-axis. We included this measure because it highlights systematic differences in feature structure between living and nonliving concepts (see Fig. 3) and is thought to reflect a core organizing principle of the conceptual system (e.g., Devereux et al., 2014; Hovhannisyan et al., 2021; Taylor et al., 2012). In our norms, living things typically have shared, highly correlated features (e.g., + *eyes*, + *legs* in ANIMAL), resulting in lower slope values (*M* = 0.67, *SD* = 0.51). Nonliving things tend to have more distinctive features that are also highly correlated (e.g., + *metal*, + *wheels* in VEHICLE), leading to higher slope values (*M* = 0.80, *SD* = 0.50; *t*(2872) = 7.00, *p* <.001).

## Results

We first report descriptive statistics for basic-level and superordinate category properties for all 260 objects, focusing on name agreement and *H*-statistic (entropy), which reflect the consistency and variability in labeling for each object. We then analyze the distribution of features generated by participants, also based on data obtained for all 260 objects. Finally, we compare our norms to existing datasets, beginning with Snodgrass and Vanderwart (1980) for basic-level labels, followed by property norm comparisons with McRae et al. (2005), the CSLB norms (Devereux et al., 2014), and Hovhannisyan et al. (2021). These comparisons are based on the objects (or object names) that are common to all norms.

### Descriptive statistics of present property norms

#### Basic-level and superordinate labels

**Fig. 4:**
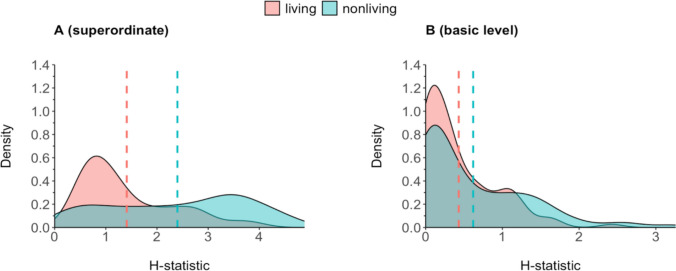
Density plots showing the distribution of *H*-statistic scores for **A** superordinate and **B** basic-level naming as a function of the objects’ living/nonliving distinction. *Dashed lines* represent the mean

For basic-level labels, the mean name agreement across all 260 concepts was 87.91% (*SD* = 13.23; range = 20-100%), and the mean *H*-statistic was 0.55 (*SD* = 0.63; range = 0.00-3.26). High consensus was observed for both living and nonliving objects, although living things consistently showed greater name agreement and lower *H*-statistics than nonliving things (see Fig. 4B). For instance, there was unanimous agreement for BANANA (name agreement = 100%; *H*-statistic = 0.00), whereas responses for less common items, such as ASHTRAY were more variable (name agreement = 60%; *H*-statistic = 2.61). This variability in naming was particularly evident for objects that are culturally outdated and less prevalent in modern contexts (e.g., spinning wheel, record player), suggesting that familiarity and cultural exposure may influence labeling consistency.

This pattern between living and nonliving things was even more pronounced for superordinate-level labels, where the mean name agreement was 59.78% (*SD* = 24.98; range = 10-100%) and the mean *H*-statistic was 2.02 (*SD* = 1.29; range = 0.00-4.89). At this level, while participants generally converged on category labels for living objects, they produced a wider variety of labels for nonliving objects (see Fig. 4A). To our knowledge, our norms are the first to report *H*-statistics for superordinate-level agreement as a function of the living/nonliving distinction, rather than solely by individual object categories. Reporting *H*-statistics in this way highlights variability in how participants assign category labels, particularly within the nonliving domain.

#### Feature distribution analysis

We also analyzed the distribution of features generated by participants, focusing on key metrics commonly reported in property norming studies (e.g., Devereux et al., 2014; McRae et al., 2005): the number of features (NOF), the number of shared features (NOsF), the number of distinctive features (NOdF), and the mean distinctiveness (MeanD) of features per concept. These measures were calculated separately for objects in the living and nonliving domains. Consistent with the McRae et al. (2005), CSLB (Devereux et al., 2014), and Hovhannisyan et al. (2021) norms, descriptive statistics for all measures were computed using a feature-frequency cutoff of 3 to reduce the influence of idiosyncratic features. These descriptive statistics are presented in Table 2.

**Table 2:** Means and standard deviations of number of features (NOF), number of shared features (NOsF), number of distinctive features (NOdF), and mean distinctiveness (MeanD) per concept for all features generated in our norms

		NOF	NOsF	NOdF	MeanD
Living	*M*	22	16.7	5.3	0.12
	*SD*	4.3	3.9	3	0.05
Nonliving	*M*	22.4	17.2	5.2	0.12
	*SD*	3.4	3.6	3.2	0.06

Results revealed that living and nonliving objects had a similar total number of features and comparable mean distinctiveness. Although these differences were not statistically significant, nonliving things elicited numerically more shared and distinctive features than living things. This pattern is partly consistent with findings from the category-specific semantic deficits literature, in which object concepts in the living domain are often characterized by a greater number of shared features due to their taxonomic organization, whereas object concepts in the nonliving domain tend to have more distinctive features, reflecting their functional properties (e.g., Clarke & Tyler, 2015; Taylor et al., 2012). The lack of significant category differences may partly reflect the response limit imposed in our norms: given that participants were restricted to producing three features per object, this constraint may have reduced variability in the NOF, NOsF, and NOdF across categories, contributing to the similarity observed between living and nonliving objects.

#### Sensorimotor feature distribution

Given the growing prominence of embodied theories of semantic representation (e.g., see Meteyard et al., 2012, for a review), we also report on the distribution of sensorimotor features across object categories in the living and nonliving domains. Using the Lancaster Sensorimotor norms (Lynott et al., 2020), we coded the dominant perceptual modality for each feature (e.g., + *yellow* as “visual”, + *sweet* as “gustatory”). The modalities included auditory, gustatory, haptic, interoceptive, olfactory, and visual.

As shown in Table 3, visual features were the most common across both domains, accounting for 71% of all features produced in our norms. However, notable domain-specific differences emerged in other modalities. For instance, nonliving objects showed greater emphasis on haptic features compared to living objects, reflecting the tactile interactions typically associated with tools and manipulable artefacts (NOF: *p* <.001; NOsF: *p* <.001). Auditory features were also more common in nonliving objects, likely due to the functional sounds they produce (e.g., + *alarm* for CLOCK; NOF: *p* =.003; NOsF: *p* =.03; NOdF: *p* =.02). Conversely, gustatory features were more prominent in living objects, consistent with their relevance to food-related concepts (e.g., + *sour* for LEMON; NOF: *p* =.02; NOsF: *p* <.001). No significant differences between domains were observed for interoceptive and olfactory features. These results reveal systematic differences in the distribution of sensorimotor features across domains, consistent with early findings by Warrington and colleagues (1983, 1984, 1987) showing that living and nonliving concepts differ in their reliance on perceptual and functional properties.

**Table 3:** Means and standard deviations of number of features (NOF), number of shared features (NOsF), number of distinctive features (NOdF), and mean distinctiveness (MeanD) per concept as a function of sensorimotor feature information

		NOF	NOsF	NOdF	MeanD
Living things					
Auditory	*M*	1.8	1.2	0.7	0.21
	*SD*	1.0	0.9	0.8	0.25
Gustatory	*M*	4.9	4.1	0.7	0.14
	*SD*	3.0	2.7	1.0	0.19
Haptic	*M*	3.2	3.0	0.3	0.05
	*SD*	2.1	1.9	0.6	0.06
Interoceptive	*M*	1.6	1.2	0.4	0.09
	*SD*	0.8	0.8	0.6	0.10
Olfactory	*M*	1.9	1.3	0.6	0.08
	*SD*	0.9	0.5	0.9	0.06
Visual	*M*	15.0	12.2	2.8	0.12
	*SD*	4.7	4.1	1.9	0.06
Nonliving things					
Auditory	*M*	3.1	1.8	1.3	0.20
	*SD*	2.6	1.8	1.5	0.16
Gustatory	*M*	3.1	1.7	1.4	0.21
	*SD*	3.1	1.7	1.7	0.21
Haptic	*M*	4.3	3.9	0.4	0.06
	*SD*	2.3	2.1	0.7	0.07
Interoceptive	*M*	1.4	1.0	0.4	0.11
	*SD*	0.8	0.7	0.6	0.19
Olfactory	*M*	1.7	1.5	0.3	0.10
	*SD*	0.9	0.9	0.6	0.08
Visual	*M*	15.9	12.7	3.2	0.12
	*SD*	4.1	3.4	2.3	0.06

### Comparisons with other norms

#### Comparisons with Snodgrass and Vanderwart

**Fig. 5:**
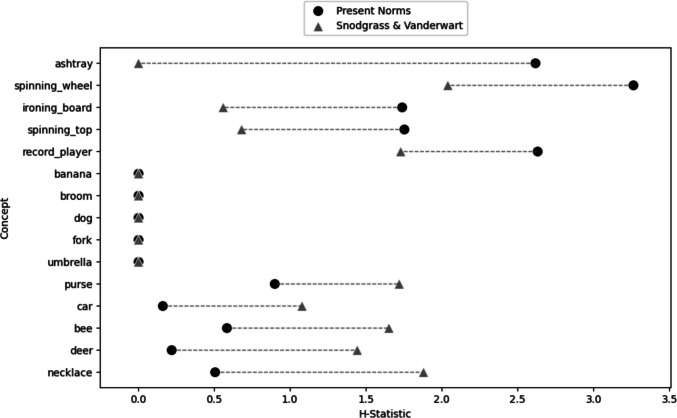
Comparison of *H*-statistic values for sample object concepts in the present norms (*black circles*) with *H*-statistic values reported for those same objects by Snodgrass and Vanderwart (*gray triangles*). The *x*-axis represents *H*-statistic values, and the *y*-axis reports the name of the selected object concepts. *Dashed lines* trace the same concept across the two norms, highlighting the difference in name agreement between the datasets. Concepts were randomly selected from three clusters based on levels of name agreement: (1) the top five objects are examples of concepts that show greater naming variability in the present norms compared to Snodgrass and Vanderwart; (2) the middle five objects have the same *H*-statistics in both datasets, indicating stable naming consensus across both datasets; and (3) the bottom five objects are examples of concepts with greater naming consensus in the present norms than in Snodgrass and Vanderwart

We compared name agreement (measured using the *H*-statistic) in our norms to that reported by Snodgrass and Vanderwart (1980) in order to assess changes in object naming over the past 45 years. On average, name agreement was similar across datasets (our norms: *M* = 0.55, *SD* = 0.63; Snodgrass and Vanderwart: *M* = 0.56, *SD* = 0.53). However, item-level analyses revealed notable differences in response patterns (*r* =.64), likely reflecting cultural shifts in object usage and familiarity over time (see Fig. 5). For instance, ASHTRAY, which showed unanimous agreement in Snodgrass and Vanderwart’s dataset, exhibited the greatest naming variability in our dataset (*H*-statistic *MDiff* = 2.61), likely due to declining familiarity with the object as a household item. Conversely, NECKLACE showed greater name agreement in our dataset than in Snodgrass and Vanderwart’s norms (*H*-statistic *MDiff* = 1.37), suggesting increased consensus in naming. Other concepts, such as DOG, remained highly conventionalized, with unanimous agreement in both datasets.

#### Comparisons with McRae, CSLB, Hovhannisyan

We compared our norms to those of McRae et al. (2005) and CSLB (Devereux et al., 2014) because they are the most widely used object property norms based on linguistic input. We also included norms from Hovhannisyan et al. (2021), which, like the present norms, were based on pictures, though their stimuli consisted of cluttered, realistic objects rather than standardized black-and-white line drawings. Comparing these datasets is crucial for understanding how different object properties may be elicited through linguistic labels versus visual depictions.

All analyses were conducted on the 157 object concepts common across all four datasets (see Appendix A for the list of object concepts, along with their corresponding names in each dataset). In order to ensure consistency, we applied a feature production frequency cutoff of five across all datasets, following the threshold used by McRae et al. (2005) and Hovhannisyan et al. (2021). We excluded Buchanan et al.’s (2013, 2019) norms from the comparisons, as their inclusion would have reduced the number of overlapping items across datasets from 157 to only 50.

##### Feature production across datasets

As shown in Table 4, systematic differences emerged between our norms and the other datasets in both the number and type of features produced per concept. Participants in our norms generated a significantly greater number of features (NOF) than those in Hovhannisyan et al. (2021), including a greater number of shared features (NOsF), but a similar number of distinctive features (NOdF). This discrepancy likely reflects differences in stimulus presentation: Hovhannisyan et al. (2021) used colored images with contextual information, which may have guided participants’ responses toward perceptual details related to the context rather than features that broadly characterize the object concept.

**Table 4:** Means and standard deviations of number of features (NOF), number of shared features (NOsF), and number of distinctive features (NOdF) per concept for our norms and those of McRae, CSLB, and Hovhannisyan. Pearson correlations (*r*) indicate the relationship between each variable in the respective norms and the present norms

Norm	Statistic	NOF	NOsF	NOdF
McRae	*M*	14.8 *	7.9 *	6.9 *
	*SD*	3.5	3.3	3.0
	*r*	0.28 *	0.26 *	0.43 *
CSLB	*M*	16.9 *	10.5	6.3 *
	*SD*	4.1	3.8	3.0
	*r*	0.29 *	0.29 *	0.44 *
Hovhannisyan	*M*	6.7 *	4.0 *	2.7
	*SD*	2.2	1.8	2.0
	*r*	0.01	0.14	0.22
Present norms	*M*	13.8	10.8	3.0
	*SD*	2.5	2.4	2.0

* Indicates significance at *p* <.001 (Bonferroni-adjusted); for *M*, significance reflects a difference from our norms (Welch’s *t* test), and for *r*, significance reflects a correlation with our norms (Pearson’s *r*)

This idea is further supported by the lack of a significant correlation in NOF and NOdF between our norms and those of Hovhannisyan et al. (2021), suggesting that participants did not consistently generate the same number of features nor the same distinctive features for a given object across datasets. While objects in our norms elicited a broad range of features, responses in Hovhannisyan et al. may have been constrained by the visual complexity or specificity of their stimuli. Their dataset yielded the lowest overall NOF, lower than both McRae et al. (2005) and CSLB (Devereux et al., 2014). This pattern suggests that participants tended to generate the same limited set of features per object, likely focusing on visually salient details in the images rather than features that generalize across exemplars. Conversely, our use of black-and-white line drawings may have encouraged participants to focus on the core conceptual representation triggered by the object, rather than surface-level visual properties. As a result, the features produced in our norms may be more generalizable and comparable to those obtained from linguistic stimuli, further supporting the use of black-and-white line drawings in property norming studies.

**Fig. 6:**
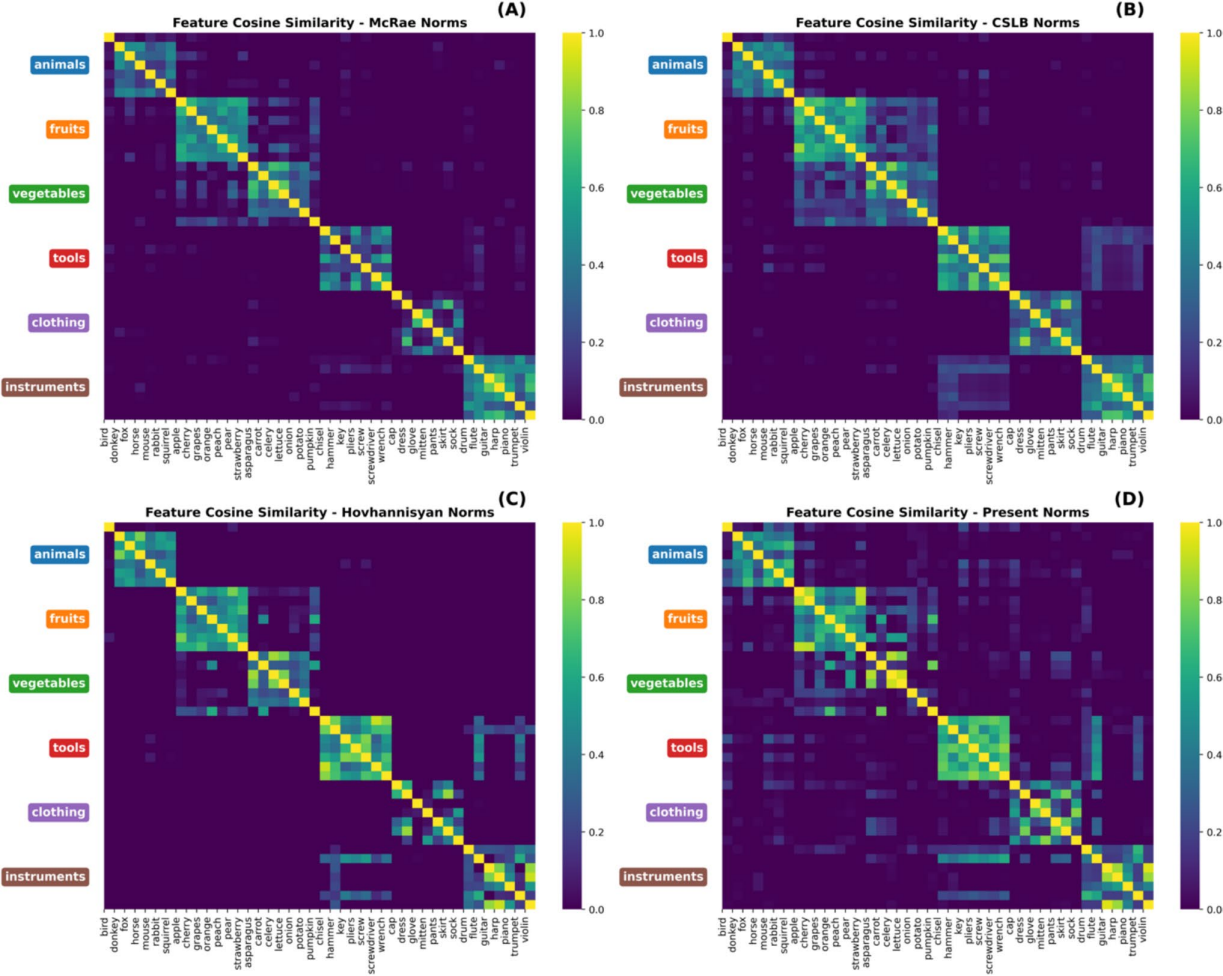
Confusion matrices of feature cosine similarity by norming dataset: **(A)** McRae et al.; **(B)** CSLB; **(C)** Hovhannisyan et al.; **(D)** Present norms. To simplify visualization, instead of displaying similarity for all 157 items, we present a subset of 42 items,randomly selecting seven items from each of the six most frequent object categories

When comparing our norms to those obtained from linguistic stimuli, different patterns emerged between the McRae and CSLB norms (see also Fig. 6). Compared to McRae et al. (2005), participants in our norms generated fewer features (NOF) but significantly more shared features (NOsF). Conversely, although NOsF did not differ between our norms and CSLB (Devereux et al., 2014), participants in our norms produced significantly fewer features in total (NOF).

Linguistic norms tended to elicit significantly more distinctive features (NOdF) than visual norms. As discussed earlier, participants in our norms primarily generated properties classified as “visual” on the sensorimotor scale (see Table 3). This reliance on perceptual features may partly account for the lower proportion of distinctive features obtained in our norms, as participants were more likely to describe generalizable visual properties rather than abstract conceptual properties. Additionally, our norms constrained participants to generate only three features per object, whereas McRae et al., CSLB, and Hovhannisyan et al. allowed for an unlimited number of responses. Thus, the response limit we imposed may have contributed to the lower NOF relative to CSLB, reduced the variability in NOF across categories (e.g., living vs. nonliving) by constraining how many properties participants could list per concept, and may have further reduced the likelihood of participants producing distinctive features.

Despite these differences, our norms were strongly correlated with both linguistic norms (see Table 4). This suggests that the concepts which elicited many features in our norms also elicited many features in the McRae et al. and CSLB norms. Moreover, although the total number of features differed, the relative distribution of shared and distinctive features was consistent across datasets, suggesting consistency in feature organization across concepts. It is also important to mention that feature-based statistics, such as distinctiveness and sharedness, are computed relative to the set of concepts included in a dataset, and thus, their absolute values can vary with the number of concepts sampled. In the present study, these statistics were calculated across all 260 Snodgrass and Vanderwart objects, following the same procedures as McRae et al. (2005) and Devereux et al. (2014). While the number of concepts included varied across norming datasets, correlations across norming studies were strong (Table 4), suggesting that the feature-based statistics obtained in the present study are comparable to those from other norming studies with larger concept sets.

##### Category structure analyses

We then analyzed category structure using both first- and second-order similarity patterns to assess how well concepts clustered within and across categories based on their feature distributions. These analyses were motivated by feature-based theories of conceptual representation, which propose that categories emerge from patterns of co-occurring features, with greater feature overlap signaling shared category membership (e.g., Moss et al., 2007; Tyler et al., 2013). Second-order analyses assessed between-category structure by computing cosine similarity matrices from concept-level feature distributions and correlating these matrices across the four norming datasets. First-order analyses assessed within-category structure by measuring the consistency of feature overlap among concepts in each category: higher similarity reflected more consistent co-occurrence of features across objects within a category, while lower similarity reflected greater variability in feature co-occurrence.

**Fig. 7:**
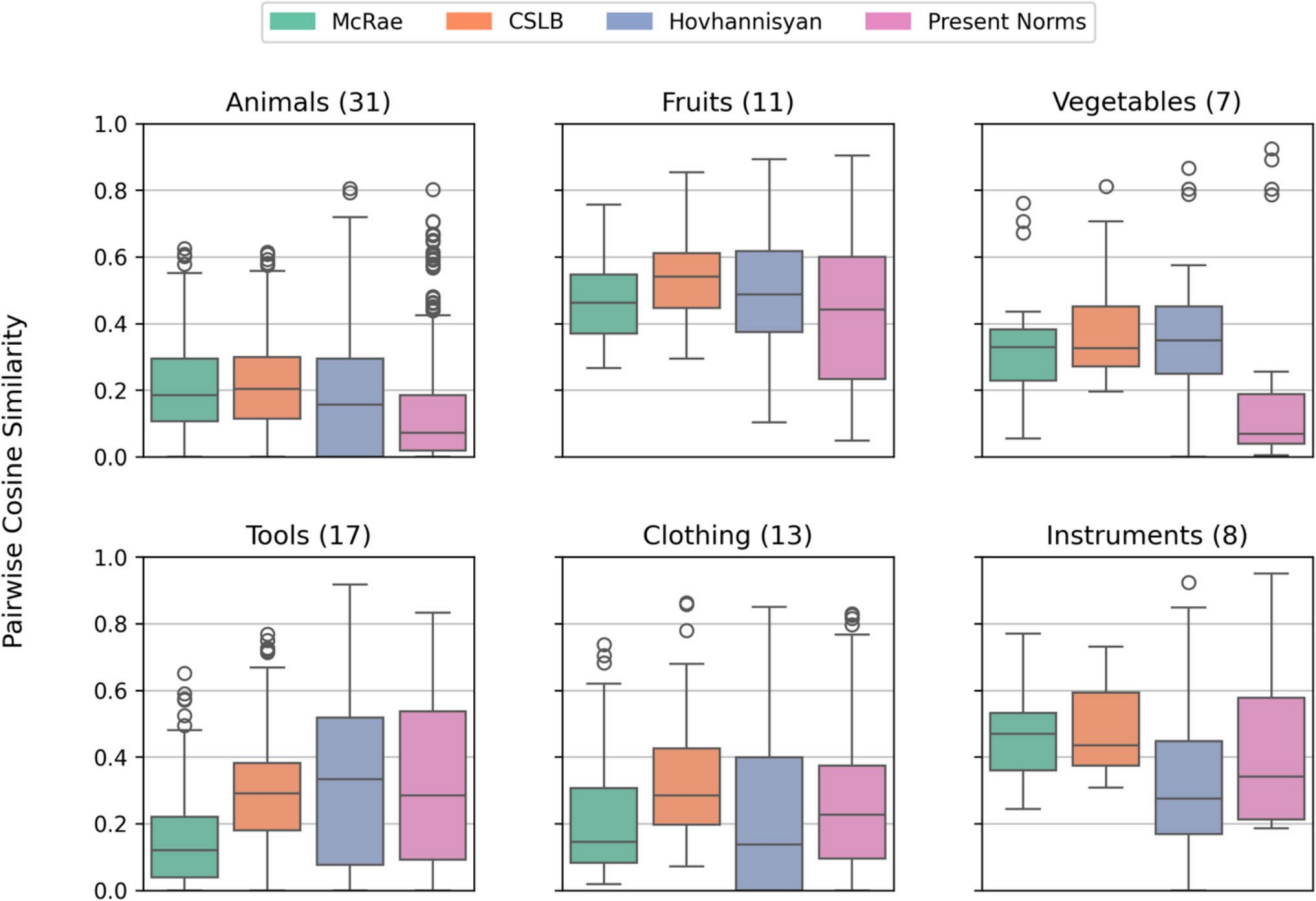
Boxplots of within-category pairwise cosine similarity across norming datasets for the six most frequent object categories. The *top panels* represent living categories, while the *bottom panels* represent nonliving categories, with the number of items in each category shown in parentheses. Higher values indicate greater feature overlap within items in a category. Wider interquartile ranges in the boxplots reflect greater variability in within-category similarity, whereas narrower ranges indicate more consistent feature overlap

Within-category similarity was quantified as the cosine similarity between feature vectors for all concept pairs. We compared similarity values across datasets using Wilcoxon signed-rank tests with Bonferroni correction for multiple comparisons. Following McRae et al. (2005) and the CSLB norms (Devereux et al., 2014), we computed cosine similarity using frequency-weighted feature vectors, where concepts were represented as rows and features as columns, with cell values indicating summed feature frequencies. Summing frequencies allowed us to quantify the strength of feature associations, rather than treating features as binary (present/absent). Results from the second-order analyses are presented in confusion matrices for each norming dataset (Fig. 6), and first-order results are shown as boxplots illustrating within-category similarity across datasets (Fig. 7).

As shown in Fig. 6, second-order similarity analyses indicated that our norms correlated most strongly with the CSLB norms (rho =.66), followed by McRae et al. (rho =.64), and Hovhannisyan et al. (rho =.60). We then conducted Fisher’s *r*-to-*z* tests to determine whether our norms were more similar to one norming dataset over another. Specifically, we compared the correlation between (a) our norms and CSLB vs. our norms and Hovhannisyan, (b) our norms and McRae vs. our norms and Hovhannisyan, and (c) between our norms and CSLB vs. our norms and McRae. Results showed that our norms were (a) significantly more similar to the CSLB than to the Hovhannisyan et al. norms (*z* = 4.25, *p* <.001) and (b) significantly more similar to the McRae et al. norms than to the Hovhannisyan et al. norms (*z* = 2.66, *p* =.02). However, (c) the correlation between our norms and CSLB did not differ significantly from that with McRae et al. (*z* = 1.59, *p* =.34).

Together, these results suggest that the overall distribution of features across categories in our norms aligns most closely with the CSLB norms, with the McRae et al. norms showing a similar, though slightly lower, degree of similarity. Conversely, the Hovhannisyan et al. norms diverged most from ours, indicating important differences in feature distribution across categories, although both were obtained through pictures of objects.

Regarding the first-order analyses, results showed that overall within-category similarity in our norms (*M* = 0.343) was closest to the CSLB norms (*M* = 0.383) and the Hovhannisyan et al. norms (*M* = 0.335) than to the McRae et al. norms (*M* = 0.287). Although these differences were not statistically significant, comparisons across individual object categories revealed distinct patterns of within-category similarity (see Fig. 7).

Across most categories, our norms showed equal or greater within-category similarity compared to the other datasets, except for the ANIMAL and VEGETABLE categories. In these two categories, our norms showed a tighter distribution of similarity values, indicating more consistent feature overlap patterns across items, although the overall average similarity was lower than in the other datasets. This suggests that, while participants consistently listed the same features for these categories, those features did not frequently co-occur across all items. For instance, in the ANIMAL category, participants consistently listed broad properties such as + *eyes* or + *legs*, but fewer features were shared across all items (e.g., + *beak* for penguins, + *fur* for some four-legged animals). Similarly, in the VEGETABLE category, participants often listed general properties like + *green* or + *stem*, but fewer features applied to all items (e.g., + *leaves* for lettuce, + *round* for onions). Thus, in both categories, while certain features were frequently mentioned, many were specific to subgroups of items rather than shared across the entire category.

When comparing within-category similarity across norms collected using visual versus linguistic stimuli, the visual norms showed a wider distribution of similarity values within categories, as indicated by larger interquartile ranges in the boxplots. This suggests that participants in the visual-based norms generated a greater variety of features across items within a given category. While some features co-occurred frequently, resulting in higher cosine similarity values in certain categories (e.g., FRUIT, CLOTHING), other sets of features were more distributed, leading to greater variability. Conversely, participants in the linguistic norms tended to list the same features more consistently, as reflected in the narrower interquartile ranges across all object categories. Moreover, within the linguistic norms, CSLB consistently showed greater within-category similarity than McRae et al., a pattern also reported by Devereux et al. (2014). This tighter category structure in the CSLB norms may be due to deliberate item selection choices, as the authors ensured that “all concepts had close semantic relatives in the set” (Devereux et al., 2014, p. 1126). This selection process likely increased within-category similarity by including concepts with closely associated neighbors. Additionally, in Devereux et al.’s norms, features were automatically standardized through synonym mapping and morphological normalization, reducing inconsistencies and limiting the number of spurious distinctive features.

Taken together, although our norms are similar to those of McRae et al. (2005) and CSLB (Devereux et al., 2014) in many respects, they provide crucial additions not available in any existing norming study. Our feature norms are the only norms based on pictures of objects that include both basic-level and superordinate properties, allowing researchers to investigate the nature of object concepts across levels of abstraction. We also provide explicit sensorimotor classifications for each feature, based on the Lancaster Sensorimotor Norms (Lynott et al., 2020). Although previous norms include many perceptual or motor features, they do not provide modality-specific coding. The McRae et al. (2005) norms include broader conceptual feature-type labels (e.g., perceptual, functional), while the CSLB and Hovhannisyan et al. (2021) norms do not include modality information. Our additions make the present norms particularly valuable for investigating modality-specific access (either via word or picture) and the role of sensory experience on conceptual access.

## From line drawings to real-world scenes: Generalizing our norms across visual stimuli

Given that our property norms were collected using black-and-white line drawings, a key question is whether the category information and features elicited from these images generalize to stimuli with greater ecological validity, such as colored drawings, realistic photographs, and photographs of objects embedded in real-world scenes. Additionally, since the Snodgrass and Vanderwart (1980) picture set contains mostly edge-based information, it was important to determine how closely our property norms align with objects under progressively more natural visual properties.

We addressed these questions by conducting a behavioral study using the same masked object (picture) and property (word) congruency task used in previous research (see Antal & de Almeida, 2024). In this task, participants are simultaneously and dichoptically presented with the image of an object and a word labeling a property of the object, and they must judge whether the property is congruent with the object. For each object, one of four types of property labels taken from our norms is shown for congruency judgment: (a) the basic-level property (e.g., *banana*), (b) a superordinate-level property (e.g., *fruit*), (c) a high-salient feature (e.g., most frequent: *yellow*), or (d) a low-salient feature (e.g., half the frequency of the high-salient feature: *peel*). In the original study, the object images consisted of Snodgrass and Vanderwart’s black-and-white line drawings. Here, we extend this work by probing object-property congruency judgments across three picture formats: (1) color-textured versions of the Snodgrass and Vanderwart line drawings (Rossion & Pourtois, 2004), (2) realistic photographs of objects in isolation, and (3) realistic photographs of objects in naturalistic scenes (see Fig. 1). Congruency judgments across these three picture formats were then compared to those obtained in Antal and de Almeida (2024) to determine whether the features elicited from black-and-white line drawings generalize to increasingly more naturalistic visual properties. Thus, if congruency agreement rates for the object-property pairs remain consistent across picture formats, this would suggest that the conceptual representations and features elicited from line drawings are generalizable to increasingly more ecologically valid object images.

This behavioral study was conducted because no existing database allows us to directly test whether our property norms generalize across picture formats. While other studies have used primed lexical decisions (e.g., Vigliocco et al., 2004; Hutchison et al., 2013; Adelman et al., 2014) or picture naming tasks (e.g., Székely et al., 2004; Brodeur et al., 2010, 2014; van Hoef et al., 2024), these tasks typically focus on lexical retrieval or object identification, rather than on object-property relations. The masked object–property congruency task offers a more direct method for probing conceptual content: by pairing each object image with one of the four probe types—basic-level labels, superordinate labels, high-salient features, or low-salient features—it is possible to determine whether the properties elicited from line drawings are generalizable across objects under more naturalistic visual conditions.

### Method

#### Participants

A total of 144 participants (132 females), between the ages of 18 and 25 (*M* = 20.12, *SD* = 1.34), were recruited from McGill University’s Psychology participant pool. All were native speakers of English (i.e., learned English before the age of 5 and used it as their dominant language), had normal or corrected-to-normal vision, were not colorblind, and reported no history of psychiatric, neurological, or cognitive impairments. Participants received one course credit in exchange for their participation. Informed consent was obtained from all participants, and procedures adhered to the ethical guidelines of McGill University’s Human Research Ethics Committee.

#### Materials

The materials were the same as those used in Antal and de Almeida (2024): 128 experimental items and 128 filler items. The 128 experimental items consisted of the Snodgrass and Vanderwart images with the highest name agreement, determined by our norming study.

In addition to the original Snodgrass and Vanderwart line drawings, we added three versions of each object image. The colored versions were taken from Rossion and Pourtois (2004), which preserve the contours and structure of the original drawings while adding color and surface texture (Fig. 1B). We also included two sets of realistic photographs: one showing the object in isolation (Fig. 1C) and another showing the same object embedded in a naturalistic scene (Fig. 1D). While Adams et al. (2024) developed a photographic version of the Snodgrass and Vanderwart line drawings, their dataset excludes 124 items. Consequently, we sourced our own realistic photographs from Adobe Stock, selecting images that matched the orientation of the original line drawings and the color scheme of the Rossion and Pourtois versions. For the isolated images, backgrounds were manually removed; in the scene version, the original photographic backgrounds were retained. Although the main goal of the present study is to provide a new set of property norms to the research community, we also make the realistic photographs of objects in isolation and embedded in naturalistic scenes available via OSF.

##### Familiarity and visual complexity of realistic images

We collected familiarity and visual complexity ratings for the realistic photographs, both in isolation and embedded in scenes, through a norming study with 80 native English speakers (F = 45, *M*_age_ = 39.7, *SD*_age_ = 14.3) recruited from Prolific. All participants met the following criteria: (a) currently residing in Canada, (b) English as both their first and primary language, (c) a Prolific approval rate between 95 and 100%, (d) no history of dyslexia or language disorders, and (e) no colorblindness.

The task was administered using Qualtrics (2015), embedded within the Prolific platform. Participants first completed a consent form and a brief language background questionnaire assessing participants’ self-reported language history and proficiency. Participants were then given instructions for the rating task, which involved judging the familiarity and visual complexity of each object on two, five-point Likert scales (1 = very unfamiliar/simple; 5 = very familiar/complex). Familiarity was defined as how often participants encounter or think about the object in daily life. Participants were asked to rate the object itself, not the way it was depicted in the picture. Visual complexity was defined as the amount of detail in the image (e.g., lines, texture), rather than the complexity of the object in the real world.

The realistic photographs were divided into two sets: objects shown in isolation and objects embedded in naturalistic scenes. Each set was further divided into four lists of 36 images, yielding a total of eight lists. Participants completed one list, and each list was rated by ten participants. Items were presented in random order and included three attention-check trials. The task began with three example trials and took approximately 15 min to complete. Participants were compensated with CAD $5 or equivalent (in UK Pounds) for a completed survey. None of the participants in the picture rating task took part in the property norming task or the congruency judgment experiment.

#### Design

Participants completed a masked object-property congruency task, using the same design and procedure outlined by Antal and de Almeida (2024), but with one modification: while the original study included both brief (60 ms) and long (200 ms) stimulus presentation durations, the current experiment used only the 200-ms presentation condition to ensure sufficient viewing time across the different picture formats.

The experiment followed a 2 × 4 factorial design, crossing (a) *stimulus lateralization* (two levels): object/left-word/right, object/right-word/left, and (b) *property type* (four levels): basic-level label, superordinate property, high-salient feature, and low-salient feature. Stimuli were divided into three sub-experiments based on picture format: (1) colored drawings (Rossion & Pourtois, 2004), (2) realistic photographs of objects in isolation (background removed), and (3) realistic photographs of objects embedded in naturalistic scenes.

In each of the three sub-experiments, the 128 experimental images were paired with each of the four property-type word labels, yielding 512 unique picture-word pairs. These pairs were crossed with the two lateralization conditions, resulting in 1024 unique experimental items per sub-experiment. These items were counterbalanced across 8 lists, such that each list contained 16 repetitions per condition, and each image appeared only once per list. Participants were assigned to one sub-experiment and completed two lists in randomized order. A total of 48 participants completed each sub-experiment, as determined through a power analysis relying on Monte Carlo simulations (80% threshold; see Antal & de Almeida, 2024).

#### Procedure and apparatus

Participants were tested individually in dimly lit rooms. Stimuli were presented on a 27-inch G-SYNC compatible Dell Alienware monitor (AW2724DM model, 2560 × 1440 resolution, 165-Hz refresh rate), connected to an Apple Mac Mini (M2 processor). Participants were positioned 60 cm from the screen via a chin and forehead rest, which ensured a constant viewing position. The size and position of the stimuli were normalized to subtend approximately 3° of visual angle.

**Fig. 8:**
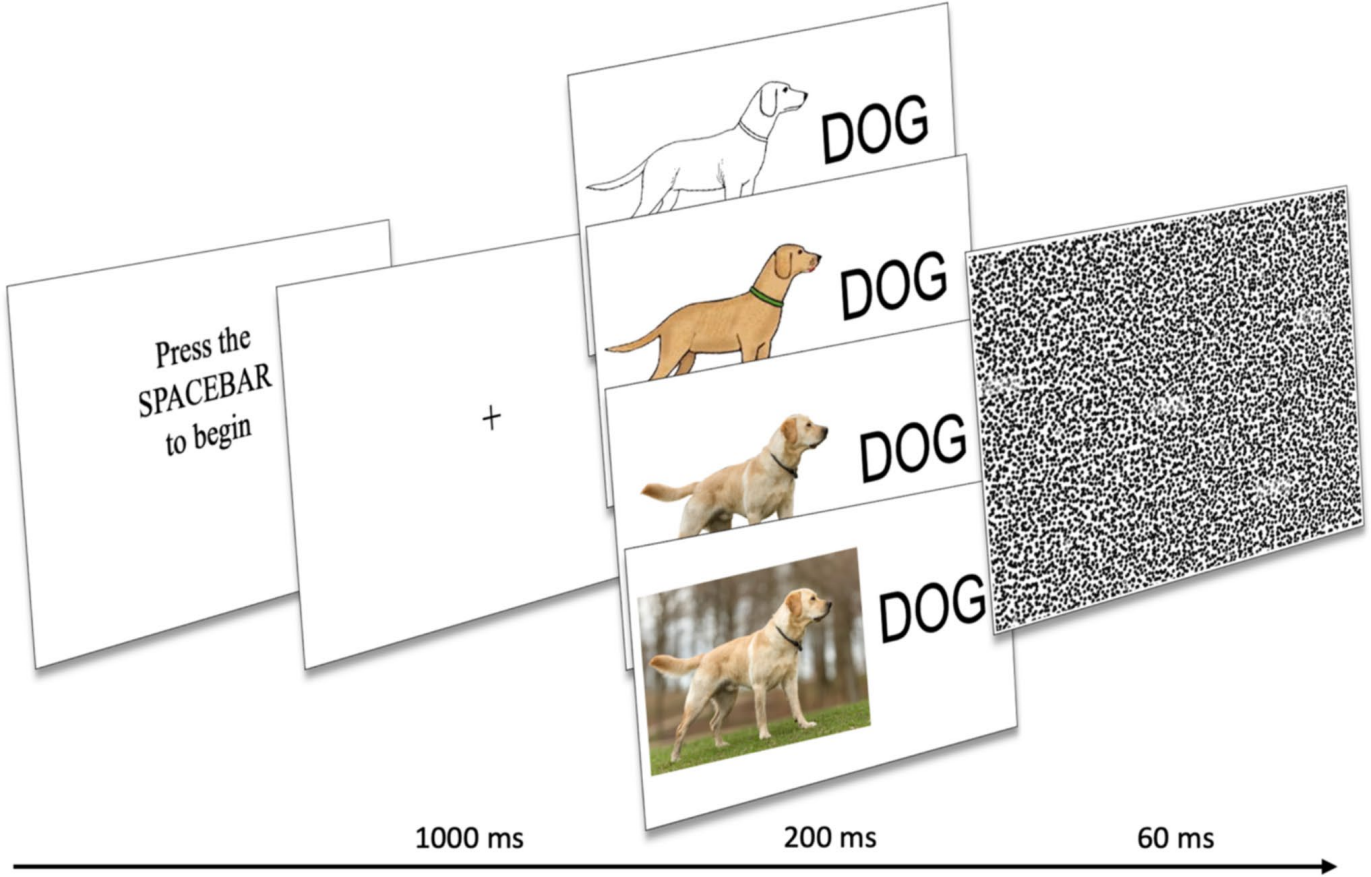
Time-course of events for each trial in the masked object–property congruency task as a function of the different picture formats. After a fixation cross, participants saw a word and a picture dichoptically, followed by a random-dot mask. Decision times and accuracy were collected from the onset of the stimuli. The different pictures of a dog, in the figure, represent different picture sets (sub-experiments). Only data from the colored line drawings (second from top to bottom), the object in isolation, and the object in the scene context were employed in the present experiment and contrasted with the results obtained by Antal & de Almeida (2024), which employed the original line drawing set (top). See text for further details

In each trial, participants were presented with a picture–word pair and were instructed to judge whether the two items were related to each other. Participants were instructed to provide their responses as quickly and accurately as possible by pressing “L” for “yes” and “A” for “no” on an Apple keyboard. The trial sequence was as follows (see Fig. 8): (1) a centrally located fixation cross appeared for 1000 ms; (2) the picture–word pair was displayed for 200 ms; and (3) a 100-ms backward mask consisting of high spatial-frequency random dots followed to prevent continued visual processing (e.g., Breitmeyer & Ogmen, 2000; Keysers et al., 2001).

The experiment began with 10 practice trials, followed by the 128 experimental items interspersed with the 128 filler items. All experimental trials required a “yes” response, while filler trials required a “no” response. Target words in the experimental trials were taken from our property norms. Filler words were not produced for the corresponding pictures in the norming study and were therefore semantically unrelated to the object (e.g., *soap* for the picture of a pumpkin).

After completing the first list, participants completed the Edinburgh Handedness Inventory (Oldfield, 1971) before proceeding to the second list. Each list lasted approximately 15 min. The experiment was programmed in PsychoPy3 (Peirce et al., 2019).

#### Data analyses

Our analyses focused on agreement rates in the congruency judgment task, defined as the proportion of “yes” responses to experimental object-property pairs. Agreement was analyzed at the trial level using binary accuracy. Response times (RTs), measured in milliseconds (ms), were analyzed at the trial level and were restricted to correct responses. The primary goal was to assess whether agreement rates for each object-property pair remained consistently high across the four picture formats: black-and-white line drawings, colored drawings, realistic photographs of objects in isolation, and realistic photographs of objects embedded in scenes.

Accuracy data were analyzed using Bayesian logistic mixed-effects models, and RT data were analyzed using Bayesian linear mixed-effects models, both fitted with the *brms* package (Bürkner, 2017) in R (Bates et al., 2013; R Core Team, 2014). All models included random intercepts for participants and items, with by-participant random slopes for stimulus lateralization and by-item random slopes for probe type (Bates et al., 2015). The basic-level probe presented with the picture on the left and word on the right served as the model baseline. Congruency judgment data for the black-and-white line drawings were taken from Antal and de Almeida (2024), using the 200-ms presentation time condition.

Fixed effects of interest included the interaction between picture format and probe type. Models also included the following covariates because they improved model fit: (a) stimulus lateralization, (b) image complexity, (c) target length, (d) number of target word repetitions per list, (e) log-transformed social usage (UCD-SD-PR), and (f) the living/nonliving category of the pictured object. All continuous predictors were centered prior to model fitting.

Weakly informative priors were used for all model parameters. Models were fit using four chains, and all parameters showed satisfactory convergence (R̂ ≈ 1).

Inference was based on posterior distributions, summarized using 95% highest-density intervals (HDI's). For accuracy models, effects are reported as odds ratios, and evidence for differences was assessed by verifying whether HDI’s excluded 1. For RT models, effects are reported as differences in milliseconds, and evidence for differences was assessed by verifying whether HDI’s excluded 0.

All participants performed above chance (i.e., above 60%) and were thus kept for all analyses. Consistent with the exclusion criteria used by Antal and de Almeida (2024), response latencies shorter than 200 ms or longer than 2500 ms (0.18% of responses) were removed as they were considered anticipations or they were deemed too long to reflect perceptual processes (see VanRullen & Thorpe, 2001). Response times analyses were conducted on correct trials only (11.41% of responses removed). Data were visualized using *ggplot2* (Wickham, 2016) in R.

### Results

#### Accuracy

**Fig. 9:**
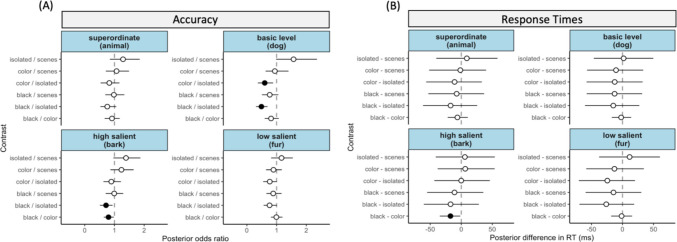
Posterior contrasts for accuracy **(A)** and response times **(B)** across picture formats. **(A)** Posterior odds ratios for accuracy, shown separately for superordinate, basic-level, high-salient, and low-salient probes. Values greater than 1 indicate higher agreement rates for the first-listed picture format in each contrast, whereas values less than 1 indicate lower agreement. The vertical reference line at 1, indicates no difference between formats. **(B)** Posterior differences in RTs (in ms), shown for the same probe types. Values greater than 0 indicate slower responses for the first-listed format, whereas values less than 0 indicate faster responses. The vertical reference line at 0 denotes no difference. *Points* represent posterior means, and *horizontal lines* represent 95% highest-density intervals (HDI’s). *Filled points* indicate contrasts for which the HDI excludes the null value (1 for odds ratios; 0 for RT differences), whereas empty points indicate contrasts whose HDI’s include the null

Overall agreement rates were high across picture formats, particularly for basic-level (96.2-98.2%) and superordinate probes (95.2-96.3%), with greater variability observed for feature-based probes (high-salient: 89.4-92.2%; low-salient: 86.3-89.3%). As shown in Fig. 9A, posterior contrasts comparing picture formats within each probe type indicated that most contrasts yielded posterior odds ratios that included 1, providing limited evidence for systematic differences in agreement across formats.

For superordinate probes, none of the contrasts between picture formats yielded HDI's that excluded 1, indicating no credible differences in agreement across formats. For basic-level probes, however, agreement was credibly lower for black-and-white line drawings than for isolated object photographs (OR = 0.49; 95% HDI [0.32, 0.70]). Agreement was also credibly lower for colored drawings than for isolated object photographs (OR = 0.60; 95% HDI [0.37, 0.89]). All remaining contrasts for basic-level probes yielded HDI’s that included 1.

For high-salient feature probes, agreement was credibly lower for black-and-white line drawings than for colored drawings (OR = 0.80; 95% HDI [0.64, 0.98]) as well as for isolated object photographs (OR = 0.72; 95% HDI [0.51, 0.94]). For low-salient feature probes, none of the contrasts between picture formats yielded HDI's that excluded 1, indicating no credible differences in agreement across formats. All remaining contrasts yielded HDI’s that included 1.

#### Response times

As shown in Fig. 9B, posterior contrasts comparing picture formats within each probe type indicated that most contrasts yielded 95% HDI’s that included 0, providing limited evidence for systematic RT differences across picture formats.

For superordinate, basic-level, and low-salient feature probes, all contrasts yielded HDI’s that included 0, indicating no credible RT differences across picture formats. For feature probes, however, there was only one noticeable difference. Namely, for high-salient feature probes, RTs were credibly faster for black-and-white line drawings than for colored drawings (β = -17.41 ms; 95% HDI [ -34.54, -1.61]). All remaining contrasts yielded HDI’s that included 0.

Overall, posterior contrasts provided limited evidence for systematic differences in congruency judgments across picture formats. Most contrasts yielded odds-ratio HDI’s that included 1, corresponding to points clustered around the midline in Fig. 9, indicating little to no difference between formats. When credible differences were observed, their magnitudes were small and close to the null value. Importantly, in all such cases, agreement was lower for black-and-white line drawings than for formats with greater ecological validity. No contrasts indicated higher agreement for black-and-white line drawings relative to the other picture formats.

Taken together, results from accuracy and RT analyses suggest that the property norms we obtained from black-and-white line drawings extend to more naturalistic stimuli. If the norms we collected were specific to the black-and-white line drawings, agreement rates should have been credibly higher for the black-and-white picture format only. Instead, when credible differences were observed, they consistently favored more naturalistic images, with lower agreement rates for black-and-white line drawings, thus supporting the generalizability of our norms to stimuli with greater ecological validity.

## Conclusions

In the present study, we introduced a new set of property norms for 260 object concepts based on the Snodgrass and Vanderwart (1980) picture set. These norms were collected from 100 native English speakers, who provided a basic-level label, a superordinate category label, and three features for each object, resulting in a dataset of 78,000 features. Our goals were threefold: (1) address the methodological limitations in existing object-concept norming studies, (2) evaluate the structure of our norms by analyzing feature statistics and comparing these statistics to existing norming datasets, and (3) evaluate whether the properties elicited from black-and-white line drawings are generalizable to stimuli with increasing ecological validity, such as colored drawings, realistic photographs of objects in isolation, and realistic photographs of objects embedded in real-world scenes.

Compared to existing property norms based on linguistic stimuli (e.g., Devereux et al., 2014; McRae et al., 2005) and those based on visual stimuli (e.g., Hovhannisyan et al., 2021), the present norms provide important methodological advances. First, we systematically and separately probed for basic-level labels, superordinate category labels, and object features, which avoided the conflation of taxonomic and conceptual features commonly found in other norms. This approach also provides a clear framework for investigating category membership, feature-based representations, and feature distributions across categories. Second, our norms were collected using free feature generation rather than constrained response formats, avoiding the unnatural feature descriptions that can arise from predefined relation labels or drop-down menus (e.g., Devereux et al., 2014; Hovhannisyan et al., 2021). Finally, with 100 participants contributing responses for each object, the present dataset includes more responses per concept than other property norming studies, substantially exceeding the 30 to 66 participants per item typical of those other studies (see Table 1; e.g., McRae et al., 2005; Devereux et al., 2014; Buchanan et al., 2013, 2019).

Crucially, this new set of norms provided the basis for the masked object-property congruency experiment reported here (see also Antal & de Almeida, 2024; Antal et al., 2025). This behavioral experiment was conducted to evaluate whether object properties, ranging from broad category labels to fine-grained features, are generalizable and consistently accessed across pictures of objects that vary in ecological validity. Object–property pairs targeting basic-level labels, superordinate properties, as well as high- and low-salient features of objects were contrasted across four picture formats: black-and-white line drawings, colored drawings, realistic photographs of objects in isolation, and realistic pictures of objects in scenes. The norms specified which properties were most likely to come to mind for each object at different levels of abstraction (i.e., superordinate, basic level, features). Thus, the norms were essential for selecting object-property pairs that would reliably tap conceptual knowledge across all picture formats and probe whether access to object properties changes under more naturalistic visual conditions. Bayesian mixed-effects models were used to assess agreement and RTs to object–property congruency judgments across picture formats.

Results showed that most contrasts yielded posterior distributions consistent with no difference between picture formats. When credible differences were observed, these differences were small in magnitude and consistently reflected lower agreement for black-and-white line drawings relative to more naturalistic pictures. This pattern suggests that properties elicited from line drawings are not restricted to that picture format and, instead, extend to stimuli with greater ecological validity.

Beyond the present study, these norms serve as a new resource for research on the nature of conceptual representations in the visual modality. The norms provide systematic data about object properties across multiple levels of abstraction, from superordinate categories to basic-level labels and object features. These norms will facilitate research in areas such as semantic memory, object recognition, language-vision interactions, and the investigation of clinical cases in neuropsychology. They also enable investigation of important questions about the nature of concepts, particularly how different perceptual conditions, attentional demands, or task constraints affect the access and retrieval of object properties. Moreover, the norms offer a valuable resource for training and evaluating computational models of conceptual knowledge, grounded in detailed categorical and semantic feature representations. Finally, given that the norms were generated using the standardized Snodgrass and Vanderwart (1980) picture set, which is widely used across cognitive and neuropsychological research, they provide a basis for reanalyzing decades of research that employed this picture set, now with reference to detailed categorical and semantic feature representations.

The norms, including raw participant responses and detailed feature statistics, are freely available. They provide a strong empirical foundation for future studies investigating how conceptual knowledge is represented and accessed across tasks, populations, and modalities. The norms will thus support a wide range of experimental and computational research in cognitive neuroscience.

## Supplementary Information

Below is the link to the electronic supplementary material.

Supplementary file1 (DOCX 26.4 KB)

## Data Availability

The study data are publicly available at https://osf.io/c6brw/overview

## Footnotes

1 We use the term “object concept” to refer to the conceptual representation underlying both a word that names an object (e.g., *dog*) and perceptual instances of that object (e.g., the referent of a dog in the real world or an image of a dog).

2 We use capital letters for concepts (e.g., DOG), italics for words or object referents (e.g., *dog*), and a “ + ” before words in italics to denote constituent features of a concept (e.g., + *bark*).

3 While there is extensive literature on abstract concepts (see Conca et al., 2021; Banks et al., 2023, for reviews), we eschew discussion of it here, as it lies outside the primary focus of the present paper. Our focus here is specifically on property norms for concrete concepts—that is, object concepts—rather than on abstract concepts or on ratings along abstract semantic dimensions of concepts such as semantic richness, arousal, emotional valence, embodiment, imageability, or affordances.

4 Color information may facilitate object recognition mainly in cases where color is highly diagnostic (e.g., Tanaka & Presnell, 1999; Antal & de Almeida, 2024; see Bramão et al., 2011, for a review).

## Code availability

The analysis scripts are publicly available at https://osf.io/c6brw/overview

## Declarations

### Open practices statements

The norms, data files, and scripts for all analyses are available on OSF (https://osf.io/c6brw/overview).
