# Supplementary material for: Semantic properties and categorization norms for the 260 Snodgrass and Vanderwart objects: A 45-year conceptual update to a classic set
Source: Behav Res Methods. 2026 Jul 8;58(8):229. doi: 10.3758/s13428-026-03069-y (PMC13346191; doi:10.3758/s13428-026-03069-y)
Supplement: Supplementary file 1 — Supplementary file1 (DOCX 26.4 KB) [file 13428_2026_3069_MOESM1_ESM.docx]

**Appendix A**

List of object concepts shared across the four norming datasets, along with their corresponding names in each dataset.

| Living things | | | |
| --- | --- | --- | --- |
| Present norms | McRae et al. | CSLB | Hovhannisyan et al. |
| ant | ant | ant | ant |
| apple | apple | apple | apple |
| asparagus | asparagus | asparagus | asparagus |
| banana | banana | banana | banana |
| bear | bear | bear | bear |
| bee | wasp | bee | bee |
| beetle | beetle | beetle | beetle |
| bird | canary | canary | canary |
| butterfly | butterfly | butterfly | butterfly |
| camel | camel | camel | camel |
| carrot | carrot | carrot | carrot |
| cat | cat | cat | cat |
| caterpillar | caterpillar | caterpillar | caterpillar |
| celery | celery | celery | celery |
| cherry | cherry | cherry | cherry |
| chicken | chicken | chicken | chicken |
| cow | cow | cow | cow |
| deer | deer | deer | deer |
| dog | dog | dog | beagle |
| donkey | donkey | donkey | donkey |
| duck | duck | duck | duck |
| eagle | eagle | eagle | eagle |
| elephant | elephant | elephant | elephant |
| fly | housefly | housefly | flying insect |
| fox | fox | fox | fox |
| frog | frog | frog | treefrog |
| giraffe | giraffe | giraffe | giraffe |
| goat | goat | goat | goat |
| gorilla | gorilla | gorilla | gorilla |
| grapes | grape | grape | grape |
| horse | horse | horse | horse |
| lemon | lemon | lemon | lemon |
| leopard | leopard | leopard | leopard |
| lettuce | lettuce | lettuce | lettuce |
| lion | lion | lion | lion |
| lobster | lobster | lobster | lobster |
| mouse | mouse | mouse | mouse |
| mushroom | mushroom | mushroom | mushroom |
| onion | onions | onion | onion |
| orange | orange | orange | orange |
| ostrich | ostrich | ostrich | ostrich |
| owl | owl | owl | owl |
| peach | peach | peach | peach |
| peacock | peacock | peacock | peacock |
| pear | pear | pear | pear |
| penguin | penguin | penguin | penguin |
| pig | pig | pig | pig |
| pineapple | pineapple | pineapple | pineapple |
| potato | potato | potato | potato |
| pumpkin | pumpkin | pumpkin | pumpkin |
| rabbit | rabbit | rabbit | rabbit |
| seal | seal | seal (animal) | seal |
| sheep | sheep | sheep | sheep |
| snail | snail | snail | snail |
| snake | rattlesnake | rattlesnake | snake |
| spider | spider | spider | spider |
| squirrel | squirrel | squirrel | squirrel |
| strawberry | strawberry | strawberry | strawberry |
| swan | swan | swan | swan |
| tiger | tiger | tiger | tiger |
| tomato | tomato | tomato | tomato |
| turtle | turtle | turtle | turtle |
| zebra | zebra | zebra | zebra |
| Nonliving things | | | |
| Present norms | McRae et al. | CSLB | Hovhannisyan et al. |
| accordion | accordion | accordion | accordion |
| airplane | airplane | aeroplane | passenger plane |
| anchor | anchor | anchor | anchor |
| ashtray | ashtray | ashtray | ashtray |
| axe | axe | axe | axe |
| baby carriage | buggy | pram | baby carriage |
| ball | ball | ball | tennis ball |
| balloon | balloon | balloon | balloon |
| barrel | barrel | barrel | barrel |
| baseball bat | bat (baseball) | bat (sporting) | baseball bat |
| basket | basket | basket | toy basket |
| bed | bed | bed | bed |
| belt | belt | belt | belt |
| bicycle | bike | bicycle | bicycle |
| blouse | blouse | blouse | blouse |
| book | book | book | book |
| boot | boots | boots | boot |
| bottle | bottle | bottle | bottle |
| bowl | bowl | bowl | bowl |
| broom | broom | broom | broom |
| brush | brush | brush | hairbrush |
| bus | bus | bus | school bus |
| cake | cake | cake | cake |
| candle | candle | candle | candle |
| cannon | cannon | cannon | cannon |
| cap | cap (hat) | cap | cap |
| car | car | car | car |
| chain | chain | chain | chain |
| chair | chair | chair | chair |
| chisel | chisel | chisel | chisel |
| cigar | cigar | cigar | cigar |
| cigarette | cigarette | cigarette | cigarette |
| clock | clock | clock | clock |
| comb | comb | comb | hair comb |
| couch | couch | sofa | couch |
| cup | cup | cup | glass |
| doll | doll | doll | doll |
| doorknob | doorknob | doorknob | doorknob |
| dress | dress | dress | dress |
| dresser | dresser | dresser | dresser |
| drum | drum | drum | drum |
| envelope | envelope | envelope | envelopes |
| fence | fence | fence | wooden fence |
| flute | flute | flute | flute |
| football | football | football | football |
| football helmet | helmet | helmet | helmet |
| fork | fork | fork | fork |
| garbage | bin (waste) | bin | trash bin |
| glove | gloves | gloves | glove |
| guitar | guitar | guitar | acoustic guitar |
| gun | gun | gun | gun |
| hammer | hammer | hammer | hammer |
| harp | harp | harp | harp |
| helicopter | helicopter | helicopter | helicopter |
| jacket | jacket | jacket | jacket |
| kettle | kettle | kettle | kettle |
| key | key | key | key |
| knife | knife | knife | knife |
| lamp | lamp | lamp | accent lamp |
| mitten | mittens | mittens | mitten |
| motorcycle | motorcycle | motorcycle | motorcycle |
| necklace | necklace | necklace | pearl necklace |
| needle | pin | needle | needle |
| pants | pants | trousers | pants |
| pen | pen | pen | pen |
| pencil | pencil | pencil | pencil |
| piano | piano | piano | piano |
| pipe | pipe (smoking) | pipe (smoking) | pipe |
| pliers | pliers | pliers | pliers |
| refrigerator | fridge | fridge | refrigerator |
| ring | ring (jewelry) | ring (jewelry) | ring |
| ruler | ruler | ruler | ruler |
| sailboat | sailboat | boat | sailboat |
| scissors | scissors | scissors | scissors |
| screw | screws | screw | screw |
| screwdriver | screwdriver | screwdriver | screwdriver |
| shirt | shirt | shirt | shirt |
| shoe | shoes | shoes | sneakers |
| skirt | skirt | skirt | skirt |
| sled | sled | sledge | sled |
| sock | socks | sock | sock |
| spoon | spoon | spoon | spoon |
| stool | stool (furniture) | stool | step stool |
| telephone | telephone | telephone | telephone |
| thimble | thimble | thimble | thimble |
| toaster | toaster | toaster | toaster |
| train | train | train | train |
| truck | truck | truck | truck |
| trumpet | trumpet | trumpet | trumpet |
| umbrella | umbrella | umbrella | umbrella |
| violin | violin | violin | violin |
| wheel | wheel | wheel | wagon wheel |
| whistle | whistle | whistle | whistle |
| wrench | wrench | spanner | wrench |
